# Integrating Physiology and Architecture in Models of Fruit Expansion

**DOI:** 10.3389/fpls.2016.01739

**Published:** 2016-11-21

**Authors:** Mikolaj Cieslak, Ibrahim Cheddadi, Frédéric Boudon, Valentina Baldazzi, Michel Génard, Christophe Godin, Nadia Bertin

**Affiliations:** ^1^INRIA/CIRAD/INRA Project-team Virtual Plants, UMR AGAPMontpellier, France; ^2^INRA PSH, Domaine Saint PaulAvignon, France

**Keywords:** functional-structural plant modeling, virtual fruit, vascular patterns, fruit shape, water and dry matter transport

## Abstract

Architectural properties of a fruit, such as its shape, vascular patterns, and skin morphology, play a significant role in determining the distributions of water, carbohydrates, and nutrients inside the fruit. Understanding the impact of these properties on fruit quality is difficult because they develop over time and are highly dependent on both genetic and environmental controls. We present a 3D functional-structural fruit model that can be used to investigate effects of the principle architectural properties on fruit quality. We use a three step modeling pipeline in the OpenAlea platform: (1) creating a 3D volumetric mesh representation of the internal and external fruit structure, (2) generating a complex network of vasculature that is embedded within this mesh, and (3) integrating aspects of the fruit's function, such as water and dry matter transport, with the fruit's structure. We restrict our approach to the phase where fruit growth is mostly due to cell expansion and the fruit has already differentiated into different tissue types. We show how fruit shape affects vascular patterns and, as a consequence, the distribution of sugar/water in tomato fruit. Furthermore, we show that strong interaction between tomato fruit shape and vessel density induces, independently of size, an important and contrasted gradient of water supply from the pedicel to the blossom end of the fruit. We also demonstrate how skin morphology related to microcracking distribution affects the distribution of water and sugars inside nectarine fruit. Our results show that such a generic model permits detailed studies of various, unexplored architectural features affecting fruit quality development.

## Introduction

The architectural properties of a fruit, such as its size, shape, internal structure (number of carpels, pericarp thickness, etc.) and pattern of vasculature can be remarkably diverse. For instance, in tomato there is a large phenotypic dissimilarity between varieties, from small round cherry tomatoes to multicarpel beefsteak tomatoes. Since water and dry matter are delivered to fruit tissues through the vasculature system, these architectural traits may have a significant impact on the distribution of water and dry matter inside the fruit, and, ultimately, on the overall development and quality. For example, vascular patterns may account for the preferential supply of metabolites to specific tissues (Moco et al., 2007), and for causing physiological disorders such as blossom-end-rot (apical tissue necrosis) and shriveling of the fruit's skin. In kiwifruit and other fleshy fruit such as grape vine (Rogiers et al., 2004), a shrivel disorder at the final stages of growth appears as a longitudinal pattern of softening and shrinkage starting at the stylar (blossom) end (Thorp et al., 2007). The cause of this disorder may be a consequence of reduced xylem density or hydraulic conductance that prevents adequate supply of water to the stylar end (Clearwater et al., 2012). At the fruit's surface, the pattern and the density of cuticular cracks (Gibert et al., 2007, 2010) together with the microclimate surrounding the fruit (Li et al., 2001; Saudreau et al., 2007) may impact water loss due to transpiration. As a consequence, the soluble solids content (sugar level) inside the fruit may change and thus ground a direct connection between the distribution of sugars inside the fruit and the biophysical properties of its skin.

Understanding the regulation of the development of fruit qualities is challenging, because they result from the interplay between physical and physiological processes that are under the control of both genetic and environmental factors. In the last 15 years, fruit quality build-up has been investigated by using process-based models that treat the fruit as one large, homogeneous compartment, neglecting the fruit's internal structure (Fishman and Génard, 1998; Bertin et al., 2006; Martre et al., 2011). To account for the fruit's shape, 3D models have been developed in the last decade. They focus on the fruit's external geometry and treat its interior as a homogeneous medium, without differentiation into tissue types (Nguyen et al., 2006; Saudreau et al., 2007; Ho et al., 2008; Mebatsion et al., 2011).

Most methods for generating 3D representations of the external shape of the fruit use equation-based techniques, such as spherical equations (Saudreau et al., 2007) or ellipsoid parametric equations (Ling et al., 2007), or reconstructions from images, such as contours extracted from several photographs of the whole fruit (Jancsok et al., 2001) or slices of the fruit (Mebatsion et al., 2011). More accurate (non-symmetric) external shape representations can be produced from X-ray CT imaging (Rogge et al., 2015). Reconstruction from images has also been used to generate some internal tissues of the fruit by manually tracing their shapes in photographs of fruit slices (Takayama et al., 2010). Such models have been mainly used to investigate fruit post-harvest behavior, for instance heat exchange in apple fruit (Saudreau et al., 2007) or mango fruit (Nordey et al., 2014), and water transport (Nguyen et al., 2006) or gas exchange in pear fruit (Ho et al., 2008), but the feedback between local properties and the resulting fruit physiology is missing. Additionally, these fruit models do not consider the vascular network that supplies the fruit with resources and how its architecture and transport properties may affect fruit growth and composition.

Bussières (1994) developed a preliminary model of water import in tomato fruit that takes into account the vascular system and predicts changes in water and dry matter content. The model assumes the fruit is spherical and the vascular pathway is a network of cylindrical filters whose ends are distributed inside the fruit. Recently, methods for generating 3D representations of fruit vasculature were introduced using magnetic resonance imaging (Moriwaki et al., 2014) or X-ray microtomography (Herremans et al., 2015). These methods produce representations that are well suited for studying the anatomy and development of fruit vasculature, but have not been incorporated into 3D simulations of fruit growth.

The aim of this work is to quantitatively investigate effects of fruit architectural properties on fruit quality by means of a 3D functional-structural fruit model. We describe a model that combines selected biophysical functions with an accurate description of fruit shape, tissue compartmentalization, and vascular networks. We use it to examine the impact of fruit structure on water and dry matter distribution in two model fruits: Tomato (*Solanum lycopersicum*), which is representative of berries, and nectarine (*Prunus persica*), which is representative of drupes. The key difference is that tomato fruit has a heterogeneous internal structure with regular skin, whereas nectarine fruit has a homogeneous interior with microcracking on its skin. Moreover, tomato has a much lower fruit conductance to water and thus it has a lower transpiration rate compared to nectarine. Using models of these two fruits, we investigate how physiological processes may be modified by specific fruit architectural properties.

In particular, we investigate the role of vascular patterns in water and dry matter distribution in tomato fruit. We test indirectly the hypothesis that reduced calcium transport to the blossom end of the fruit via xylem vessels (assumed to be proportional to xylemic water flow; Bar-Tal et al., 1999) causes the calcium-deficiency disorder blossom end rot (BER) (Belda and Ho, 1993; Ho and White, 2005). By modifying vascular patterns in our model, we further test if increasing the size of bundles and producing a more regular distribution of them helps to prevent BER (similarly to treating plants with abscisic acid; de Freitas et al., 2011). We then test the hypothesis that the spatial pattern of cuticular microcracks on nectarine fruit changes the rate of water loss due to transpiration and results in a high fruit soluble solids concentration under the part of the skin with high microcrack density (Wu et al., 2003).

**Figure 1:**
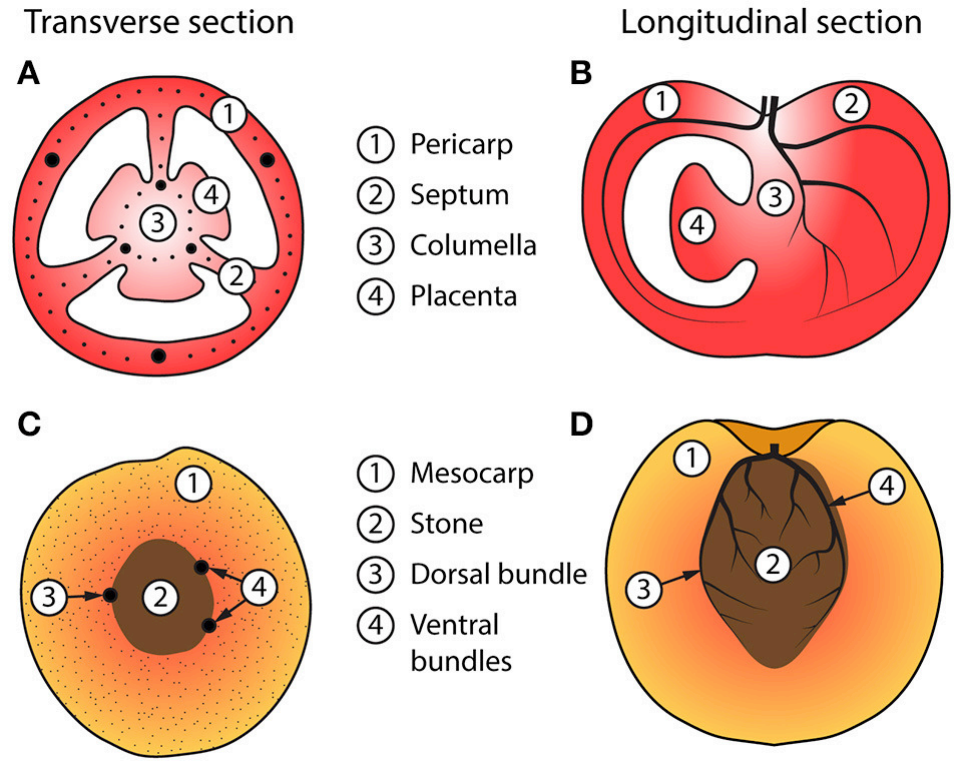
**Schematic of idealized fleshy fruits showing various tissues**. A transverse section **(A)** and longitudinal section **(B)** of a tomato fruit with three carpels, showing the pericarp, columella, placenta, and vascular bundles. The vascular bundles are further divided into those formed in the pericarp and those in the fruit axis (columella, placenta, and septum). Similarly, a transverse section **(C)** and longitudinal section **(D)** of a nectarine fruit, showing the mesocarp, stone, and vascular bundles. Ventral and dorsal bundles surround the stone and secondary branching bundles occupy the mesocarp [in **(D)** shown as large and small black dots, respectively].

**Figure 2:**
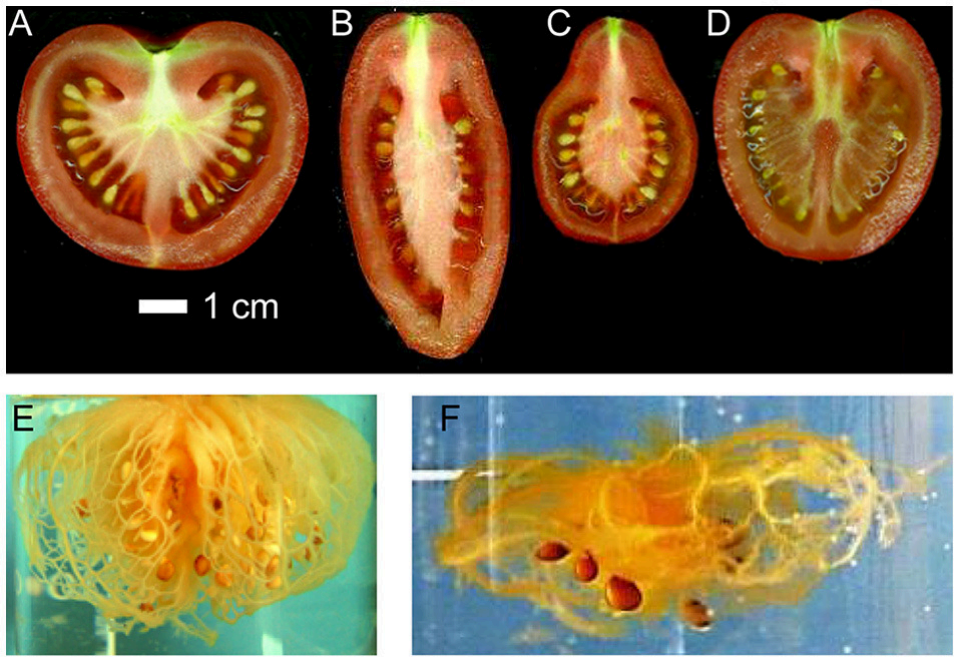
**Diversity of fruit architecture in tomato**. The images show longitudinal sections of four tomato varieties with different external shapes and internal structures: **(A)** round, **(B)** elongated, **(C)** obovoid, and **(D)** ellipsoid. Additional images show the vascular networks in **(E)** Levovil and **(F)** Cervil varieties. These different architectures have a role in the development of fruit quality.

We restrict our approach to the phase where fruit growth is mostly due to cell expansion (cell division has stopped) and the fruit has already differentiated into different tissue types. The main fruit tissues that we represent in the model are in the case of tomato: (1) pericarp and septum (formed from the ovary wall of the flower), (2) columella and placenta, to which seeds are attached, and (3) the vascular system, which transports water, carbohydrates, and nutrients to the other tissues. In the case of nectarine, the main fruit tissues are (1) mesocarp, (2) stone, and (3) the vascular system. We base our representation on anatomical descriptions of fruit, and selected tomato (Kanahama et al., 1990) and nectarine (Zhang et al., 2009) as primary examples to emphasize the variability of fruit anatomy, see Figure 1. We then incorporated the main physiological processes that are involved in the balance of water and dry matter flows between the fruit, plant, and environment, as these drive fruit growth. Our intention was to represent the diversity of fruit architectures, such as in tomato fruit (Tanksley, 2004; Rodriguez et al., 2011) (see Figure 2), and to integrate these digital representations with the physiological processes that modulate fruit quality development.

## Materials and methods

**Figure 3:**
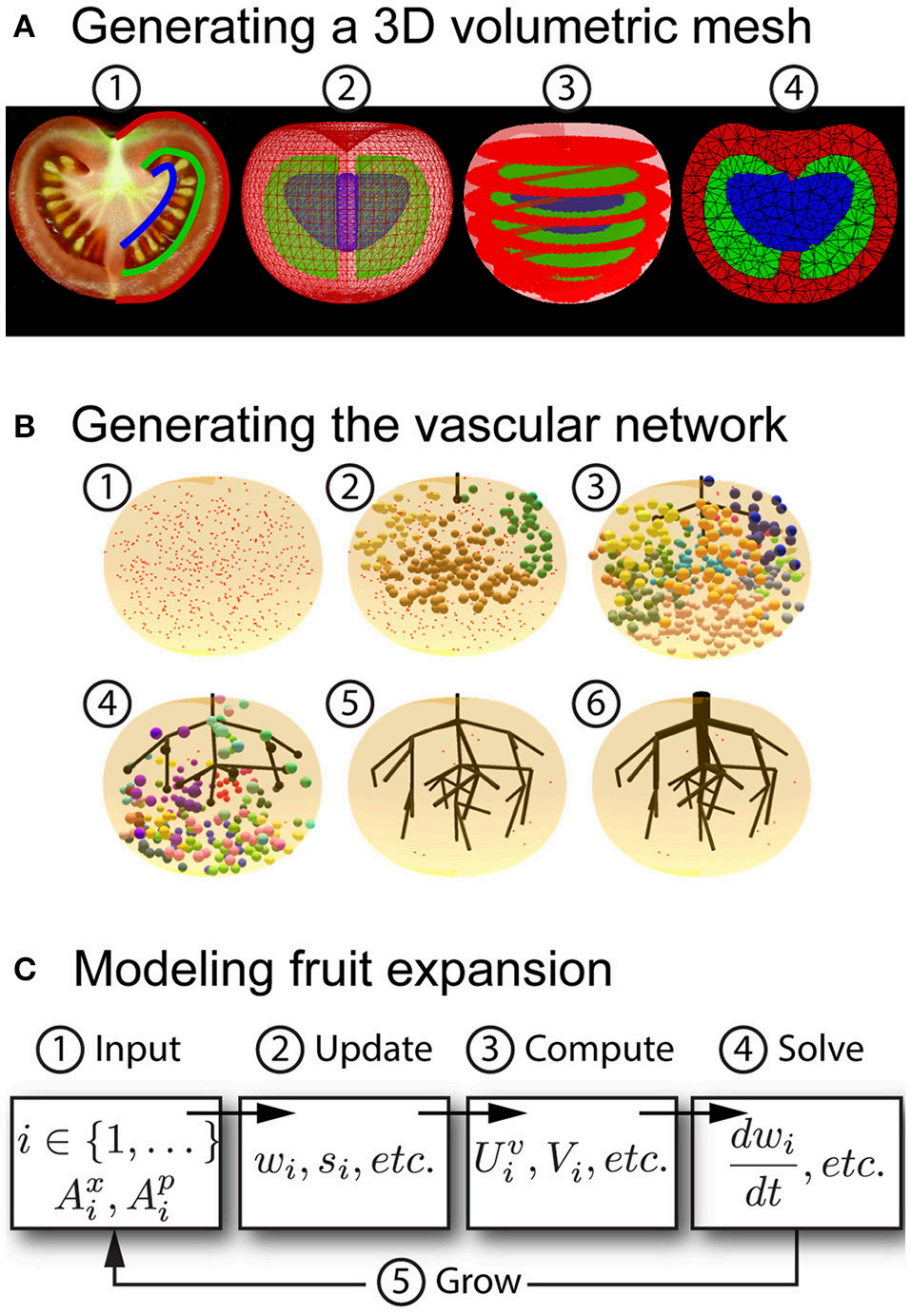
**Overview of the modeling pipeline. (A)** Steps in generating a 3D volumetric mesh of a fruit. (1) User-defined 2D curves are traced from an image. (2) Polyhedral surfaces are generated from the curves. (3) A stack of segmented images are generated from the surfaces. (4) A tetrahedral mesh is generated from the image stack. **(B)** Steps in creating vascular geometry inside a 3D mesh. Initial step (1): Attractor points (red squares) are placed randomly within the volumetric mesh to signify the availability of space for new vascular bundles. Subsequent steps (2–5): New bundles are added into the vascular network based on the neighborhood of attractor points (highlighted by colored spheres) that are sensed by the tips of existing bundles (black spheres). A different color is assigned to the neighborhood of points that is sensed by each developing bundle. In (2), for example, brown spheres are the points that are sensed by the tip of the main bundle, whereas yellow and green spheres are sensed by two laterals. At every step the highlighted attractor points are removed after the creation of a new bundle. Final step (6): The radii of vessels are set according to Murray's law, which relates the radii of daughter branches to the radii of the parent branch. **(C)** Steps in simulating fruit expansion. (1) Input information is extracted from the mesh and vascular geometry, namely, the tetrahedral compartments and contact surface areas of vessels in those compartments. (2) State variables are updated, such as water and dry matter content. (3) Flows of water and dry matter content are computed. (4) The system of coupled ordinary equations is solved. (5) Contact surface areas and mesh geometry are updated. The process is repeated until a final simulation time is reached.

To construct a functional-structural fruit model, we developed a modeling pipeline in the OpenAlea platform (Pradal et al., 2008) that involves three steps: (1) creating a 3D volumetric mesh representation of the internal and external fruit structure, (2) generating a complex network of vasculature that is embedded within this mesh, and (3) integrating aspects of the fruit's function, such as water and dry matter transport, with the fruit's structure. Figure 3 gives an overview of the steps we developed in this modeling pipeline.

### Generating a 3D volumetric representation of fruit structure

**Figure 4:**
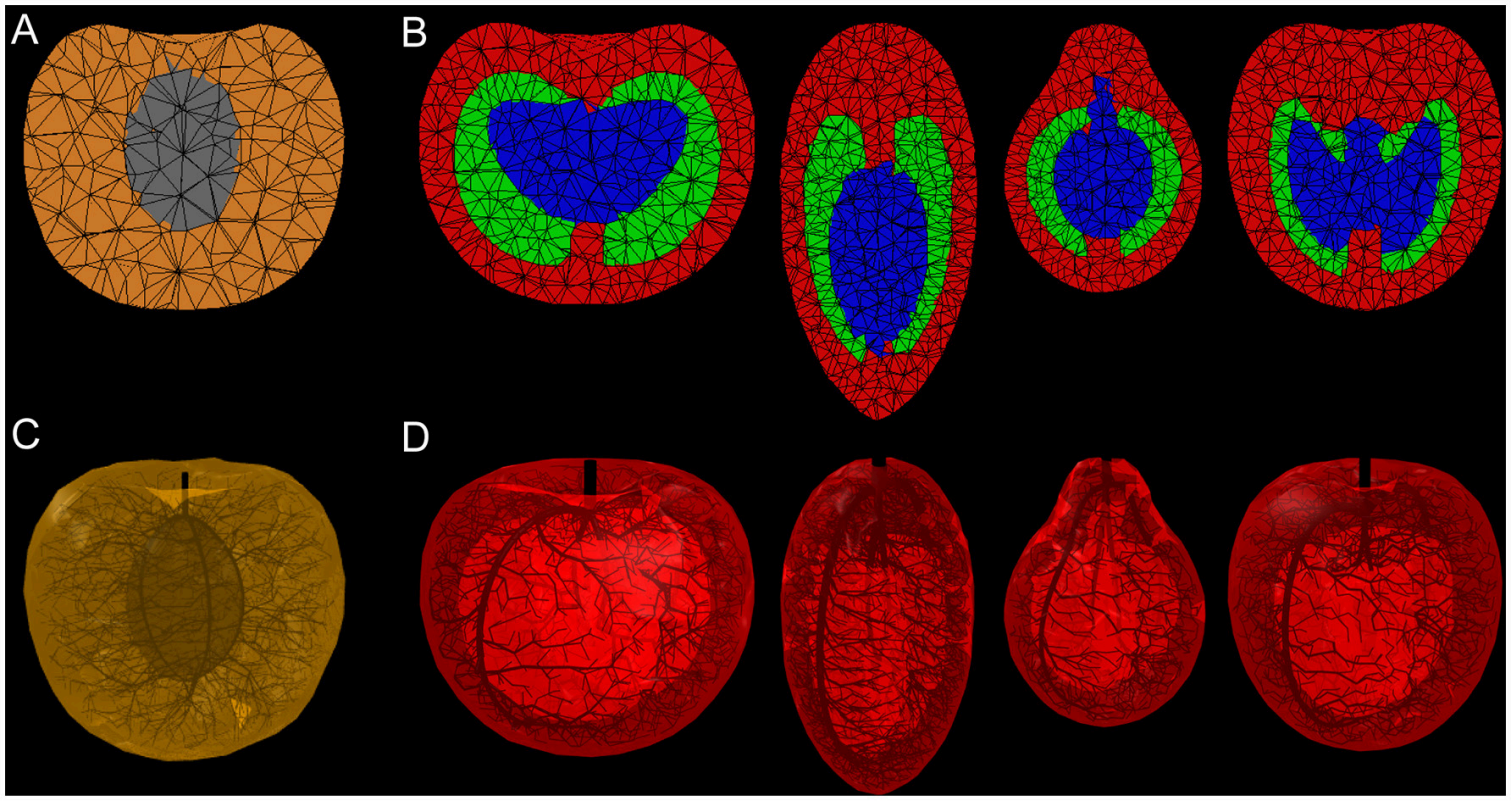
**Longitudinal sections through 3D meshes of fruit (A,B)** and generated vascular networks **(C,D)**. The images show the results of applying our mesh generation procedure to create 3D volumetric representations of **(A)** nectarine and **(B)** tomato fruit. Each mesh was generated from user-defined 2D curves that were traced from images (e.g., the four tomato varieties shown in Figure 2). For nectarine **(C)**, the main ventral and dorsal vascular bundles on the endocarp were defined by the user, whereas the branching secondary bundles were generated algorithmically. For tomato **(D)**, the large vascular bundles of the pericarp and fruit axis (columella, placenta, and septum) were generated by the user, whereas secondary bundles were generated algorithmically. The properties of these vascular networks can be easily modified, e.g., by increasing the number of large vascular bundles in the pericarp (see Figure 6).

Figure 3A illustrates the different steps of our pipeline for generating a 3D volumetric mesh of a fruit. We used the reconstruction algorithm of the 3D mesh generation package from the Computational Geometry Algorithms Library, CGAL, which is a C++ library offering many geometric algorithms and data structures (Alliez et al., 2011). For our purposes, the reconstruction algorithm in CGAL is used to generate a 3D tetrahedral representation of a fruit from two forms of input: (1) a 3D polyhedral surface, which is useful when the internal structure of the fruit is relatively simple, or (2) a 3D image, which captures the internal and external structure of the fruit. The polyhedral surface can be generated by profile sweeping of 2D curves using algorithms from the PlantGL library (Pradal et al., 2009), where the curves can be either user-defined or derived from images of perpendicular slices of a fruit. The 3D image can be generated algorithmically from several user-defined polyhedral surfaces that represent various tissues in the fruit, which can once again be constructed using the profile sweeping algorithms in PlantGL. Since each surface defines a boundary of a fruit tissue, the space between surfaces can be used to identify tissue types. For example, in a model of stone fruit, one surface can be used to represent the skin and another to represent the stone. The space between these surfaces identifies the mesocarp. Thus, each point in the 3D image is labeled according to its position relative to these surfaces. For instance, label 0 identifies regions outside of all surfaces (not forming part of the fruit), label 1 between the skin and internal tissues (such as the mesocarp), and label 2 the innermost tissue (such as the stone). The resulting 3D image is given as input into the CGAL reconstruction algorithm, which uses a generator based on Delaunay refinement with a mesh optimization phase. The CGAL reconstruction algorithm accepts parameters defining a lower bound on the angle between surface facets of a tetrahedron, upper bounds on the size of surface facets, and upper bounds on the size of internal tetrahedra. With these parameters, CGAL's mesh generation allows us to have fine control over the accuracy and quality of the volumetric mesh (the size and number of tetrahedra is chosen as a compromise between the accuracy and complexity of the simulation). Figure 4 shows the results of applying our technique to generate meshes of nectarine and tomato fruit (shown in Figure 2). This illustrates the flexibility of our pipeline for modeling a large variety of fruit architectures.

### Generating a 3D representation of vascular tissue

It is difficult to use image-based methods for generating a representation of the fruit's vascular tissues because of the small size of vascular bundles relative to other tissues. The control of vascular pattern formation in fruit is not well understood (de Freitas et al., 2011). However, mechanistic models have been proposed in the context of leaf vein formation (Rolland-Lagan and Prusinkiewicz, 2005), and suggest ways to address the construction of vessel architecture in fruits. Here, we adapted an algorithmic approach inspired by the Space Colonization Algorithm (SCA) that was originally proposed for synthesizing leaf venation patterns (Runions et al., 2005) and extended to more complex 3D branching structures (Runions et al., 2007). To allow for a flexible control over the vessel architecture inside different fruit tissues, we implemented the SCA in the L-system based plant modeling platform L-Py (Boudon et al., 2012). The vascular geometry can be generated from descriptions of observed vascular patterns in a fruit (e.g., using graphically-defined contours to specify the main bundles). In our model, the branching structure of the vascular bundles is represented as a tree structure made of a set of vascular segments connected to each other by edges whenever two segments are adjacent. The main veins of these vascular networks may be sketched with user-specified control curves representing the shape of large vascular bundles, such as the ventral and dorsal bundles on the endocarp of stone fruit. Secondary vascular bundles are generated algorithmically by the SCA. During the construction process, a set of points is homogeneously distributed in 3D space to serve as attractors for growing the current vascular network. Based on the attraction of these points, the L-system model iteratively adds up new vascular segments to the current vascular network, thus progressively colonizing the space marked by the attractor points (Figure 3B).

To set the radii of the vessels, we apply Murray's law that relates the radius of vessels before and after a branching point (Murray, 1927): If *r* denotes the radius of a bundle supporting two branching bundles with radii *r*_1_ and *r*_2_, then we have *r*^*n*^ = $r_{1}^{n}$ + $r_{2}^{n}$, for some real number *n* that usually ranges from 1 to 4 (McCulloh et al., 2003), and that generally depends on the species. The radius of each vascular bundle is thus computed recursively from the tips of the bundles, which are assumed to have a fixed radius, basipetally to the pedicel (root node of the graph). During this basipetal process, at each junction point between segments, the radius of the parent bundle is computed according to Murray's law. Figures 4C,D show the results of generating the vascular tissue for tomato and nectarine fruit. Each vascular bundle is represented by a cylinder in a tree-like structure. The volumetric mesh was integrated with the vascular geometry by computing the lateral surface area of vasculature contained within each tetrahedron *i*. We denote this value as $A_{i}^{v}$ with *v* = *x* or *v* = *p* for xylem or phloem vessels, respectively.

### Incorporating water and dry matter transport in the 3D fruit model

We integrated a model of water and dry matter transport in the 3D volumetric mesh representing the fruit's architecture by extending a previously developed process-based model of fruit growth (Fishman and Génard, 1998; Liu et al., 2007). The fruit is represented as multiple spatial compartments instead of a single abstract compartment. Each compartment corresponds to a tetrahedron, which represents regional collections of cells within the fruit. Each tetrahedron can exchange water and nutrients with its neighbors, with the vasculature that crosses it or with the atmosphere (air) if it is at the surface of the fruit. In addition, we assume that the flow of water and nutrients is much faster within the vascular bundles compared to the flows between the vascular bundles and the fruit compartments or between the fruit compartments themselves. We thus consider that the concentration of sugars and the water potential in the vascular bundles are constant and equal to their values in the pedicel.

#### Definitions and notations

We will index the variables describing the state of each compartment (tetrahedron) by subscript *i*. For example, variables *w*_*i*_ and *s*_*i*_ represent respectively the water content and dry matter content of compartment *i*, expressed in grams. Adjacent compartments *i* and *j* may exchange water according to their relative water potential Ψ_*i*_ and Ψ_*j*_. In our fruit system, water potential Ψ_*i*_ in a compartment *i* is mainly determined as a balance between hydrostatic and osmotic pressures: Ψ_*i*_ = *P*_*i*_ − π_*i*_, where *P*_*i*_ denotes the hydrostatic pressure in compartment *i* and π_*i*_ its osmotic pressure. We know that water flows from regions of high to low water potential: high hydrostatic pressure tends to push water out of the compartment and, conversely, high osmotic pressure tends to attract water into the compartment. Hydrostatic pressure *P* depends on changes in the volume of a compartment and on the elastic and plastic growth properties of the fruit (Lechaudel et al., 2007). Osmotic pressure depends on the osmotically active compounds in the compartment, and is calculated according to van't Hoff's formula, π = *MRT*, where *M* is the molar concentration of the compounds, *R* is the ideal gas constant and *T* is the temperature (Nobel, 1974). The flow of water *U*_*ij*_ (g/h) between two compartments *i* and *j* is computed on the basis of the difference of water potential between the two compartments (Fishman and Génard, 1998):

(1) $$U_{ij}=A_{ij}L\left(\Psi_{i}-\Psi_{j}\right)=A_{ij}L\left(P_{i}-P_{j}-\left(\pi_{i}-\pi_{j}\right)\right)$$

where *A*_*ij*_ is the area separating the compartments, and *L* is the hydraulic conductivity between compartments (assumed to be constant). In case the membrane between the two compartments is not fully impermeable to the solutes, a coefficient of reflection σ is used (Fishman and Génard, 1998):

(2) $$U_{ij}=A_{ij}L\left(P_{i}-P_{j}-\sigma \left(\pi_{i}-\pi_{j}\right)\right),$$

where σ = 0 (resp. σ = 1) if the membrane fully permeable (resp. impermeable) to the osmolites.

#### The vascular compartments

The vascular tissue obtained as described in Section “Generating a 3D representation of vascular tissue” is subdivided in two twin networks that represent the xylem and phloem. The properties of the xylem and phloem in terms of osmotic and hydrostatic pressures may vary in space and time, for instance the water potential in the xylem is known to vary from the top of a plant to its roots, and depends on the hour of the day. Because our model is at a smaller (fruit) scale, we neglect spatial variations inside the vascular network, and assume that water and solute flows in vessels are fast compared to flows from vessels to fruit tissues. Hence, we model the xylem and phloem as a functionally different but single structural compartment that interacts with the fruit compartment, depending on the lateral surface area of vessels inside the compartment and the permeability to solutes. In the xylem vessels we neglect the osmotic pressure, compared to the hydrostatic pressure, i.e., setting π^*x*^ = 0. Hence the xylem water potential reduces to Ψ^*x*^ = *P*^*x*^. In addition, we assume that the lateral water flow between xylem and phloem is fast such that their water potentials are equal (Ψ^*p*^ = Ψ^*x*^). Therefore, the hydrostatic pressures in the phloem and the xylem are related by *P*^*p*^ = *P*^*x*^ + π^*p*^.

#### Rate of volume change of a compartment

Consider a compartment *i* with volume *v*_*i*_. The compartment is made of a physical envelope (e.g., the apoplastic matrix made up by all the cell walls of the compartment) and by fluids (water and solutes) that are enclosed in this envelope. A change in volume of compartment *i* is always connected by two phenomena: (1) a change in matter and (2) a deformation of the envelope. First, the change of volume is necessarily accompanied with a movement of matter, inwards or outwards (because of incompressibility of fluids). If the volume increases, matter must enter the compartment to fill in the space freed internally by the growth. Symmetrically, if the volume decreases, matter must be expelled from the compartment. In our model, we assume that the rate of volume change for each tetrahedral compartment results from the rates of change in water mass and in dry matter mass only (Fishman and Génard, 1998):

(3) $$\frac{dv_{i}}{dt}=\frac{1}{D_{w}}\frac{dw_{i}}{dt}+\frac{1}{D_{s}}\frac{ds_{i}}{dt},$$

where *D*_*w*_ and *D*_*s*_ are the densities of water and dry matter, respectively. Note that, in many cases the dry matter contribution can be neglected compared to the water term.

Second, the change in volume of the compartment must also correspond to a deformation of the envelope. In general, this deformation can be of either elastic and/or plastic (Lockhart, 1965; Ortega, 1985; Lechaudel et al., 2007). At the cell scale, the corresponding tension in the walls balances the pressure forces that arise from difference in turgor between cells; if no other force is considered, this mechanical equilibrium yields a relationship between turgor and deformation that depends on the geometry of the cells. In Lockhart/Ortega's model (Ortega, 1985; Lechaudel et al., 2007), assuming cylindrical cell geometry, this relationship is explicit, and the volume variation of each cell is expressed as a function of pressure and its variation. In this study, we use the same phenomenological model and relate the volume variation of each compartment to pressure and pressure variation by the following equation:

(4) $$\frac{dv_{i}}{dt}=\frac{v_{i}}{\epsilon }\frac{dP_{i}}{dt}+\phi v_{i}\theta \left(P_{i}-Y\right),$$

where θ(*x*) = max(0, *x*), ϕ is cell wall extensibility, ϵ is the elastic modulus of the envelope, and *Y* is the threshold pressure above which plasticity occurs. In this equation, the first term on the right-hand side denotes the elastic contribution to the deformation, while the second term corresponds to the plastic contribution.

The two preceding equations make explicit two facets of the change in volume of a compartment: On the one hand a change of volume must correspond to a change of *content* in the compartment (inflow or outflow of matter). On the other hand, the change also impacts the *container* which is stretched or compressed by the change. Both phenomena are coupled and must be consistent with each other as the volume of the container and the volume of the content must be identical at every instant. Equations (3) and (4) underline the symmetric role of the content or container changes in driving growth. Growth for example may be triggered by an increase of osmotic pressure in the compartment, creating a water influx (Equation 3) and an associated volume increase. Simultaneously, this either builds up an elastic deformation of the envelope (and thus an increase of its turgor pressure) or a plastic deformation of its envelope (or both). Reciprocally, a decrease in yield threshold may lead to a volume increase and results in a water influx to accommodate the volume change.

#### Fluxes contributing to water change in a compartment

In a fruit compartment *i*, water content may change due to different fluxes. Let $U_{i}^{v}$ be the net flux from the vessels to the compartment, *U*_*ji*_ the flux from compartment *j* to a neighboring compartment *i* (not in the vasculature), and *T*_*i*_ the transpiration flux of the compartment (if applicable), then:

(5) $$\frac{dw_{i}}{dt}=U_{i}^{v}+\sum_{j\in N(i)}U_{ji}-T_{i}$$

where *N*(*i*) is the set of the neighboring compartments of *i*.

##### Net flux of water from vessels to compartment

The first term $U_{i}^{v}$ has two subcomponents: water can flow from the vessels crossing the compartment either from the xylem ($U_{i}^{x}$) or from the phloem $\left(U_{i}^{p}\right)$,

(6) $$U_{i}^{v}=U_{i}^{x}+U_{i}^{p}$$

and the corresponding fluxes:

(7) $$U_{i}^{x}=A_{i}^{x}L^{x}\left(P_{i}^{x}-P_{i}+\pi_{i}\right)$$

(8) $$U_{i}^{p}=A_{i}^{p}L^{p}\left(P_{i}^{p}-P_{i}-\sigma^{p}(\pi^{p}-\pi_{i})\right)$$

where $A_{i}^{x}$ and $A_{i}^{p}$ are the lateral surface areas of the membrane separating the vascular bundles of xylem and phloem, respectively, from the fruit flesh, *L*^*x*^ and *L*^*p*^ are the radial hydraulic conductivities from xylem and phloem, respectively, into the fruit flesh, σ^*p*^ is the reflection coefficient of the semi-permeable membrane separating phloem and tissue in the compartment. As stated earlier, we have neglected here the osmotic pressure in the xylem.

The values of the parameters for *L*^*x*^, *L*^*p*^, and σ^*p*^ are taken from the literature and depend on the fruit species (Fishman and Génard, 1998; Liu et al., 2007). The lateral surfaces $A_{i}^{x}$ and $A_{i}^{p}$ are computed for each compartment from the actual surface area of the vascular bundles intercepted by the tetrahedral compartment and regularly updated according to growth [see Equation (24)]. The osmotic pressures inside the vascular bundles, π^*p*^, and the fruit flesh, π_*i*_, are calculated as for the sugar concentration *S*_*i*_ and *S*_*p*_, respectively in the fruit flesh and in the phloem (Fishman and Génard, 1998):

(9) $$\pi_{i}=\pi_{b}+RT\frac{S_{i}}{M_{s}}$$

and

(10) $$\pi^{p}=\pi_{b}^{p}+RT\frac{S^{p}}{M_{s}}$$

where π_*b*_ and $\pi_{b}^{p}$ are user defined base osmotic pressures, and *M*_*s*_ is the molecular mass of transported or stored sugars. The sugar concentration (expressed in g soluble sugars per g fresh weight) in the fruit flesh is calculated as:

(11) $$S_{i}=\;\frac{\beta s_{i}}{\beta s_{i}+w_{i}}$$

where β is the ratio of soluble sugars in the carbohydrate pool. The sugar concentration in the phloem is assumed to be constant and equal to the sugar concentration in the stem (see Section “The vascular compartments”).

##### Water exchanges between compartments

The water flow from compartment *j* to compartment *i*, outside of the vasculature, is proportional to the difference in water potential between the two compartments:

(12) $$U_{ji}=A_{ji}L\left(\Psi_{j}-\Psi_{i}\right),$$

where *L* is hydraulic conductivity outside of vasculature. Note that if Ψ_*i*_ > Ψ_*j*_ then the flow *U*_*ji*_ from *j* to *i* is negative, meaning that water is flowing out of compartment *i*.

##### Transpiration

The loss of water due to transpiration *T*_*i*_ for an external compartment *i* is proportional to the local difference of humidity between the fruit and the ambient air:

(13) $$T_{i}=A_{i}^{ext}\rho_{ext}\alpha \left(T\right)\left(H_{air}-H_{f}\right),$$

where $A_{i}^{ext}$ is the area of the tetrahedral compartment that is in contact with the air, ρ_*ext*_ is a permeation coefficient of the fruit surface to water vapor, α(*T*) is a coefficient modulated by temperature, *H*_*f*_ is the relative humidity in the air-filled space within the fruit, and *H*_*air*_ is the relative humidity of the ambient atmosphere. The area of an external face $A_{i}^{ext}$ is computed directly from the 3D mesh while the remaining parameters are user defined. The coefficient ρ_*ext*_ is a function of the fruit's age and is set according to stomatal and cuticular components of the fruit surface conductance.

We use an extended form of Equation (13) in the case of modeling transpiration due to microcracking (e.g., in our nectarine simulations). The rate of each external tetrahedral compartment is modeled as the sum of two rates (dependent on the cuticular and microcrack surface areas):

(14) $$T_{i}=A_{i}^{ext}\left(c_{i}\rho_{crk}+\left(1-c_{i}\right)\rho_{ext}\right)\alpha (T)(H_{f}-H_{air}),$$

where *c*_*i*_ is the fraction of $A_{i}^{ext}$ that has cuticular cracks, and ρ_*crk*_ and ρ_*ext*_ are coefficients of permeation of the fruit surface to water vapor for cuticular cracking and non-cracking, respectively. For cuticular cracks, we assume the permeation coefficient ρ_*crk*_ is constant over time. The fraction, *c*_*i*_, of cuticular cracks for each external tetrahedral compartment at time *t* is modeled according to the following equation:

(15) $$c_{i}\left(t\right)=\;\frac{z_{i}}{\sum_{j}z_{j}A_{ext,j}\left(t\right)}\cdot C\left(t\right)\sum_{j}A_{ext,j}\left(t\right)$$

where *z*_*i*_ is the percentage of cracks on tetrahedron *i*. The total cuticular crack surface area per fruit surface area, *C*(*t*), is modeled as a function of the fruit fresh weight (Gibert et al., 2007):

(16) $$C\left(t\right)=\;\{\begin{array}{cc} {\left(b\left(X^{\lambda }-a\right)\right)}^{1/\lambda } & \text{if }X^{\lambda }\geq a\\ 0 & \text{​​​if }X^{\lambda }<a \end{array}$$

where *C*(*t*) is the cuticular crack surface area per fruit surface area (%), *X*(*t*) is the fruit fresh weight (g), and *a, b*, and λ are parameters. For our nectarine simulations, the percentage of cracks per tetrahedron, *z*_*i*_, is set from our measurements at 140 dafb, and *a* = 5.396, *b* = 0.635, and λ = 0.41 values were taken from the work of Gibert et al. (2007).

#### Dry matter variation within a compartment

To complete the computation of the terms involved in Equation (11), we must evaluate the rate of variation of dry matter within the fruit compartment (*ds*_*i*_/*dt*). This rate is modulated by four processes (Fishman and Génard, 1998): bulk flow of sugars transported by water through vessel membranes ($V_{i}^{m}$), active transport of sugars from the phloem ($V_{i}^{a}$), passive diffusion due to differences in sugar concentrations ($V_{i}^{d}$), and respiration (*R*_*i*_):

(17) $$\frac{ds_{i}}{dt}=V_{i}^{m}+V_{i}^{a}+V_{i}^{d}-R_{i}$$

##### Bulk flow of sugars

As membranes may be semi-permeable to solutes, a certain amount of sugars are advected by water flow from phloem tissues to fruit. The amount depends on water flow intensity (when σ_*p*_ < 1) and its direction:

(18) $$V_{i}^{m}=\{\begin{array}{cc} \left(1\;-\;\sigma_{p}\right)U_{i}^{p}S^{p}\; & \text{ if }U_{i}^{p}\geq 0\\ \left(1\;-\;\sigma_{p}\right)U_{i}^{p}S_{i}\; & \text{ if }U_{i}^{p}<0 \end{array}$$

##### Active transport of sugars

We assume sugars are actively transported from the phloem based on Michaelis-Menten kinetics:

(19) $$V_{i}^{a}=A_{i}^{v}\nu_{max}\frac{S^{p}}{K_{m}+S^{p}}\frac{1}{1+\text{exp}((t-t^{*})/\tau )}$$

where ν_*max*_ is the maximal rate of active transport per unit area $A_{i}^{v}$ (*cf*. Goeschl et al., 1976), *K*_*m*_ is the Michaelis-Menten constant, and t^*^ and τ are time dependent parameters modeling the decline in $V_{i}^{a}$ as the fruit ages. Our equation is similar to the one used by Fishman and Génard (1998) except that the rate depends on vascular surface area instead of dry matter content. Because of this difference, we ensure that compartments with no vessels ($A_{i}^{v}$ = 0) do not actively transport sugars.

##### Passive diffusion

We assume that a contribution of sugar transport from the phloem is due to passive diffusion:

(20) $$V_{i}^{d}=A_{i}^{v}k\left(S^{p}-S_{i}\right)$$

where $A_{i}^{v}$ is the contact surface between a compartment *i* and its vessels, and *k* is a diffusion coefficient related to solute permeability.

##### Respiration

The dry matter usage *R*_*i*_ is dependent on temperature and is the sum of growth respiration, which is proportional to the dry matter variation, and maintenance respiration, which is proportional to dry matter content (Fishman and Génard, 1998):

(21) $$R_{i}=q_{g}\frac{ds_{i}}{dt}\;+\;q_{m}\left(T\right)s_{i},$$

where *q*_*g*_ is the growth respiration coefficient, and *q*_*m*_(*T*) is a function of temperature which defines the intensity of maintenance respiration.

### Change in size of tetrahedral compartments

#### Modeling growth without constraints

We implemented a simplified model of growth for small time steps by assuming that the size of the model compartments (tetrahedra) does not change significantly. At each time step *t* of the simulation, water content, dry matter content and volume changes are evaluated for each compartment *i* from Equations (3) and (4). The computed volumes *v*_*i*_(*t* + Δ*t*) are used to assess the new areas of the four faces $A_{i}^{k}\left(t+\Delta t\right)$, where *k* = {1,2,3,4} indicates the *k*-th face of a tetrahedron, and

(22) $$A_{i}^{k}(t+\Delta t)=A_{i}^{k}\left(t\right){\left(\frac{v_{i}\left(t+\Delta t\right)}{v_{i}(t)}\right)}^{2/3}.$$

The 2/3 exponent uniformly scales these faces by the change in the tetrahedron's volume. Next, contact surfaces between neighbors are estimated. Because two adjacent compartments *i* and *j* may have different growth rates, their adjoining faces $A_{i}^{k}$ and $A_{j}^{l}$ may be of different sizes. We thus define the contact surface between these adjacent faces as the minimum of the two values:

(23) $$A_{ij}\left(t+\Delta t\right)\;=\;\min\left\{A_{i}^{k}\left(t+\Delta t\right),A_{j}^{l}(t+\Delta t)\right\}.$$

The contact area between vessels and each compartment *i* is estimated by assuming that it grows proportionally to the total area of the compartment:

(24) $$A_{i}^{v}\left(t+\Delta t\right)=a_{i}^{v}A_{i}(t+\Delta t),$$

where $A_{i}=\underset{k}{\sum }A_{i}^{k}\;$ and *v* = *x* or *v* = *p* and

(25) $$a_{i}^{v}=\frac{A_{i}^{v}(0)}{A_{i}(0)}$$

The initial values $A_{i}^{v}\left(0\right)$ and *A*_*i*_(0) are defined to balance the relative contributions of xylem and phloem to fruit growth. These values are then used in Equations (3) and (4) to proceed with the following time step.

#### Modeling growth with constraints

In the simplified model of growth, we did not take into account mechanical constraints on the expansion of compartments, so faces of neighboring compartments could be of different sizes. The fundamental solution would be to compute an equilibrium state, where actual growth is a result of local growth and the mechanical properties of the tissue (Kennaway et al., 2011; Boudon et al., 2015). We implemented an approximate solution instead. After the local potential growth rate is computed (*dv*_*i*_/*dt*), we apply a global optimization procedure to adjust the vertices of the mesh so that the volume $v_{i}^{*}$ of tetrahedron *i* in the mesh matches its potential volume *v*_*i*_.

Our global optimization procedure is performed in two steps. We first scale the entire mesh by the sum of potential volumes, and then move the vertices of the mesocarp (but not the vertices of the skin and stone) to satisfy $v_{i}^{*}\approx v_{i}$. For the second step, we use Metropolis dynamics to optimize the positions of the vertices [see Corson et al. (2009) and Merks et al. (2011)]. We define a potential energy function (*H*) that balances the fruit's volumetric expansion: $H=\lambda_{v}\underset{i}{\sum }{\left(v_{i}^{*}-v_{i}\right)}^{2}$, where λ_*v*_ sets a tetrahedral compartments resistance to compression or expansion. The function *H* is minimized by iteratively moving random vertices in the mesh and accepting the move if it leads to a decrease in the value of *H*. To avoid local energy minima, moves that slightly increase the value of *H* are accepted with a small probability, given by the Boltzmann distribution: *P*(Δ*H*) = exp(−Δ*H*/*G*), where *G* is a parameter controlling the amount of moves accepted this way. A vertex is moved in a random direction as follows: $x^{*}=x+\epsilon \overset{⇀}{r}$, where *x* is the position of the vertex, ϵ is a user-defined step size and $\overset{⇀}{r}=\left[\rho \text{cos}\left(\theta \right),\;\rho \text{sin}\left(\theta \right),\;\mu \right]$, with random variables μ ∈ [−1, 1], θ ∈ [0, 2π], and $\rho =\sqrt{1-\mu^{2}}$. Once all of the vertices have been moved (whether or not the move was accepted), we check if the value of *H* has dropped below a minimum threshold. If it has not, another iteration of the Metropolis dynamics is performed until there has been no change in the value of *H* over a user-specified number of iterations (at least ten in our case). Since this second step does not guarantee that $v_{i}^{*}=v_{i}$, we assume water can be quickly redistributed within the fruit. Consequently, we adjust the water content in each compartment by setting $w_{i}=\left(v_{i}^{*}-\frac{s_{i}}{D_{s}}\right)D_{w}$.

### Model implementation

The unknown variables of the problem are, for each tetrahedron *i*, the water content *w*_*i*_, the dry matter content *s*_*i*_, and the pressure *P*_*i*_; their time evolution is described by Equations (5), (17), and (4), respectively, while the volume *v*_*i*_ is unambiguously defined from *w*_*i*_ and *s*_*i*_ by Equation (3). The initial value problem defined by these coupled ordinary differential equations is solved using the backward differentiation methods from ODEPACK (Hindmarsh, 1983), which is wrapped by the Python package SciPy (Jones et al., 2001).

### Experimental measurements

#### Tomato measurements

The vascular pattern of a tomato fruit (cv Levovil 120 g fresh weight) was observed after pealing the fruit and dislocating the pericarp by microwaving the fruit in water at low power. The fruit was slightly stirred and rinsed into distilled water to remove the residues until we obtained the skeleton of the main vessels (Figures 2E,F).

#### Nectarine measurements

The heterogeneity of sugar content was measured within the flesh of ripe nectarine fruit, cv Magique (200 g fresh weight). The fruit flesh was divided into 4 longitudinal slices and each slice was cut radially into 8 pieces of equivalent size. Each of these 8 pieces was divided in 2 parts, internal (stone side) and external (skin side). Then total soluble solid contents (Brix index) were measured in the 64 small flesh volumes with a refractometer (ATAGO PR32 Alpha). In peach, this index strongly correlates with the soluble sugar content of the flesh fresh matter, as shown by Grechi et al. (2008) over several years of experimentation. Three repetitions were performed for each part of the flesh. The skin of the external regions was removed, died with toluidine blue (0.1%v/v) in order to stain cuticular cracks dark blue for image analysis. The image analysis procedure from Gibert et al. (2007, 2010) was followed using *ImageJ* software instead of the *ImageTool* program.

## Results

Our virtual fruit model is intended to be generic and to capture a variety of fruit architectures either of fleshy or stone fruits. In this section, we first test the ability of the model to reproduce the architecture of observed tomato fruits with various shapes and vascular networks. We analyze the main impact of these architectures on sugar and water transport using simulations and test the consistency of the model outputs from different perspectives. We then demonstrate the ability of the model to be used in applied agronomic contexts. To this end, we construct a functional-structural model of a nectarine fruit and use it to study the effect of skin microcracking on the development of fruit quality. We thereby test *in silico* the experimental results of Wu et al. (2003) on peach fruit, which indicated a high fruit soluble solids concentration of the flesh situated under the part of the skin with high microcrack density.

### Assessing the ability of the model to represent different shapes and vascular architectures

We first tested the ability of our model to account for different shapes and architectures of fruits. We examined various geometries of tomato fruits with different shapes, and generated 3D models using the procedure presented in Section “Materials and Methods” (Figure 3). The vascular networks in the pericarp tissue were generated with the algorithm presented in Section “Generating a 3D representation of vascular tissue.”

The tomato mesh had 2547 total tetrahedra (of which 1615 represented the pericarp), with an average volume of 1.2 × 10^−2^ cm^3^ and s.d. 4.1 × 10^−3^ cm^3^. The vascular network was initiated with a section of peduncle and ten bundles positioned at equal distances from each other in the pericarp and in the direction of the fruit's main axis. The remaining vessels were computed by the space colonization algorithm. The parameters of the vascular model were adjusted so that the density was of the same order of magnitude as presented by Belda and Ho (1993) for a similar fruit size. The order of magnitude of the density was also in agreement with measurements on larger tomato sizes (de Freitas et al., 2011). The diameters of the vessels were computed according to Murray's law, with different values of the pipe exponent *n* = {1,2,3,4}, and a fixed value of the stem diameter (at the pedicel junction). This exponent has an impact on how the vessel diameters decrease with branching; for instance, in the case of the division of one vessel of radius *r*_0_ in two vessels of equal radii *r*_1_, Murray's law implies: $r_{0}^{n}=2r_{1}^{n}$, and, therefore, $r_{1}=2^{-1/n}\;r_{0}$. Consequently, *r*_1_ = *r*_0_/2 when *n* = 1, and *r*_1_ = *r*_0_ when *n* tends to infinity.

**Figure 5:**
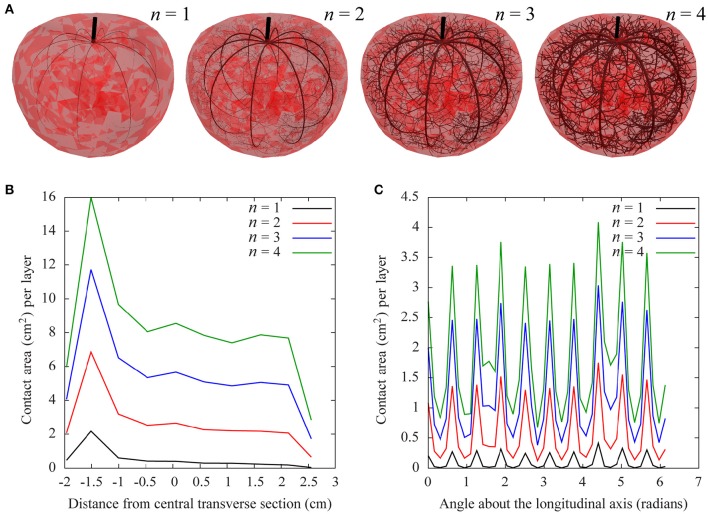
**Example of a functional-structural tomato fruit model. (A)** 3D visualization of the vascular networks in tomato for different values of the pipe exponent (*n* = 1.2,3,4), with the same pedicel diameter. **(B,C)** Total surface area of vessels in different parts of the tomato fruit, for different values of the pipe exponent: **(B)** in ten transversal layers, given by the distance from the central layer: –2 cm (pedicel) to 2.5 cm (blossom end), and **(C)** in 40 longitudinal sections, given as the angle (in radians) from a reference angle. The peaks correspond to the angular positions of the thick primary bundles.

Figure 5 shows a 3D representation of these vascular networks on a tomato fruit and quantitative measurements of their properties. The fruit is divided into 10 transversal layers and 40 longitudinal sections pivoting the pedicel to blossom end. In each of these layers, the total contact area between the vessels and the fruit is computed for each value of the pipe exponent. The 3D representation (Figure 5A) shows that vessel diameters of child branches decrease more slowly compared to parent branches as the pipe exponent increases (following Murray's law), but the vessel density (number of vessels per layer) remains the same. Figure 5B shows that for these values of the pipe exponent, the contact area decreases from the pedicel to blossom end, with a plateau in the middle of the fruit and a sharp decrease at the blossom end. The total contact area increases linearly with the pipe exponent (data not shown). Figure 5C shows 10 peaks in contact area, which correspond to the positions of the main vascular bundles. The intensity of these peaks increases with the pipe exponent, as well as the value in between the peaks. For a given value of the pedicel diameter, the tissues in between vascular bundles are better vascularized when the pipe exponent is higher.

**Figure 6:**
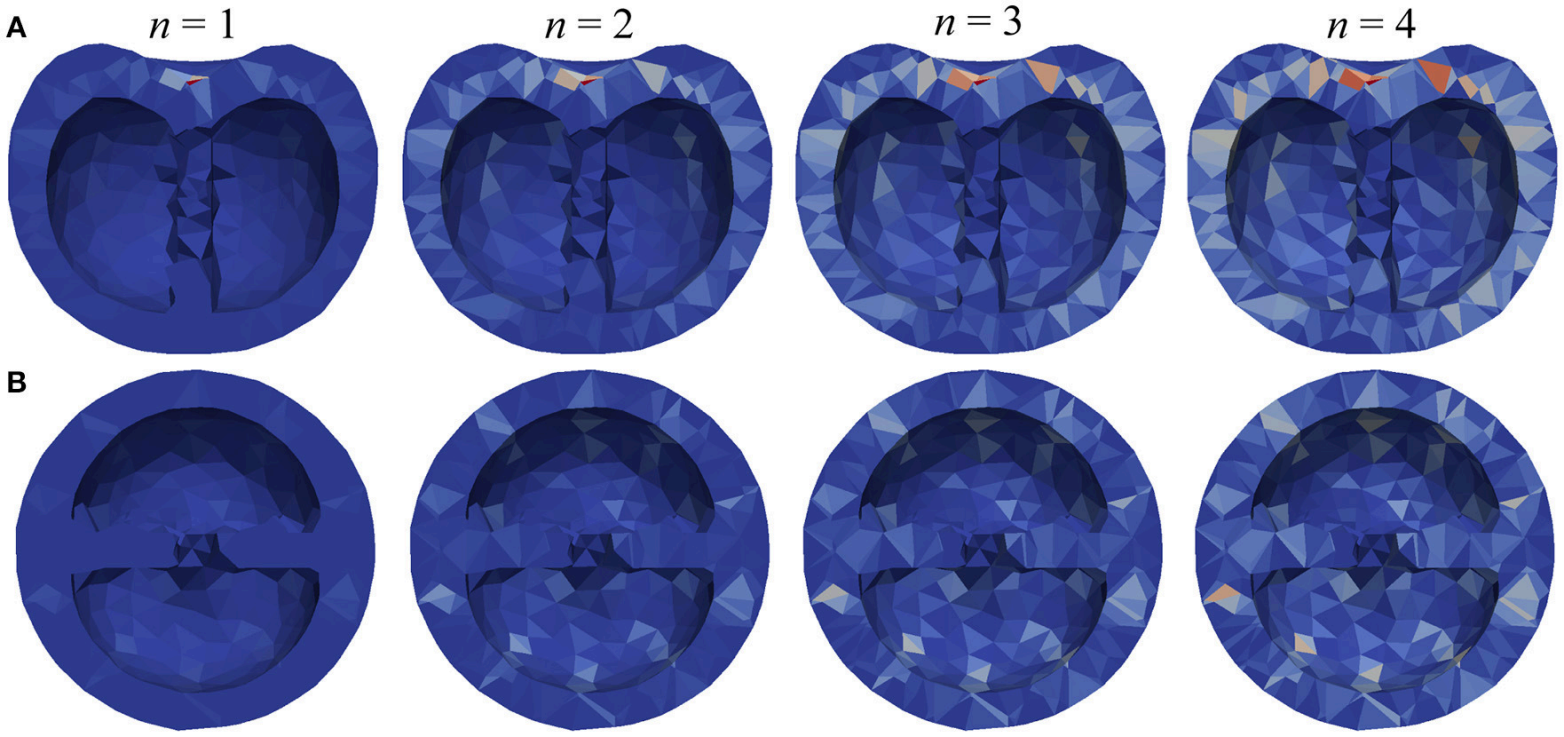
**Visualizations of the relative water mass variation in tomato over a 24 hour period. (A)** Longitudinal and **(B)** transverse sections are shown for different values of the pipe exponent (*n* = 1.2,3,4). Each tetrahedron is colored according to its change in water content using the same color map: dark blue indicates 0% change and red indicates 2% change per hour.

**Figure 7:**
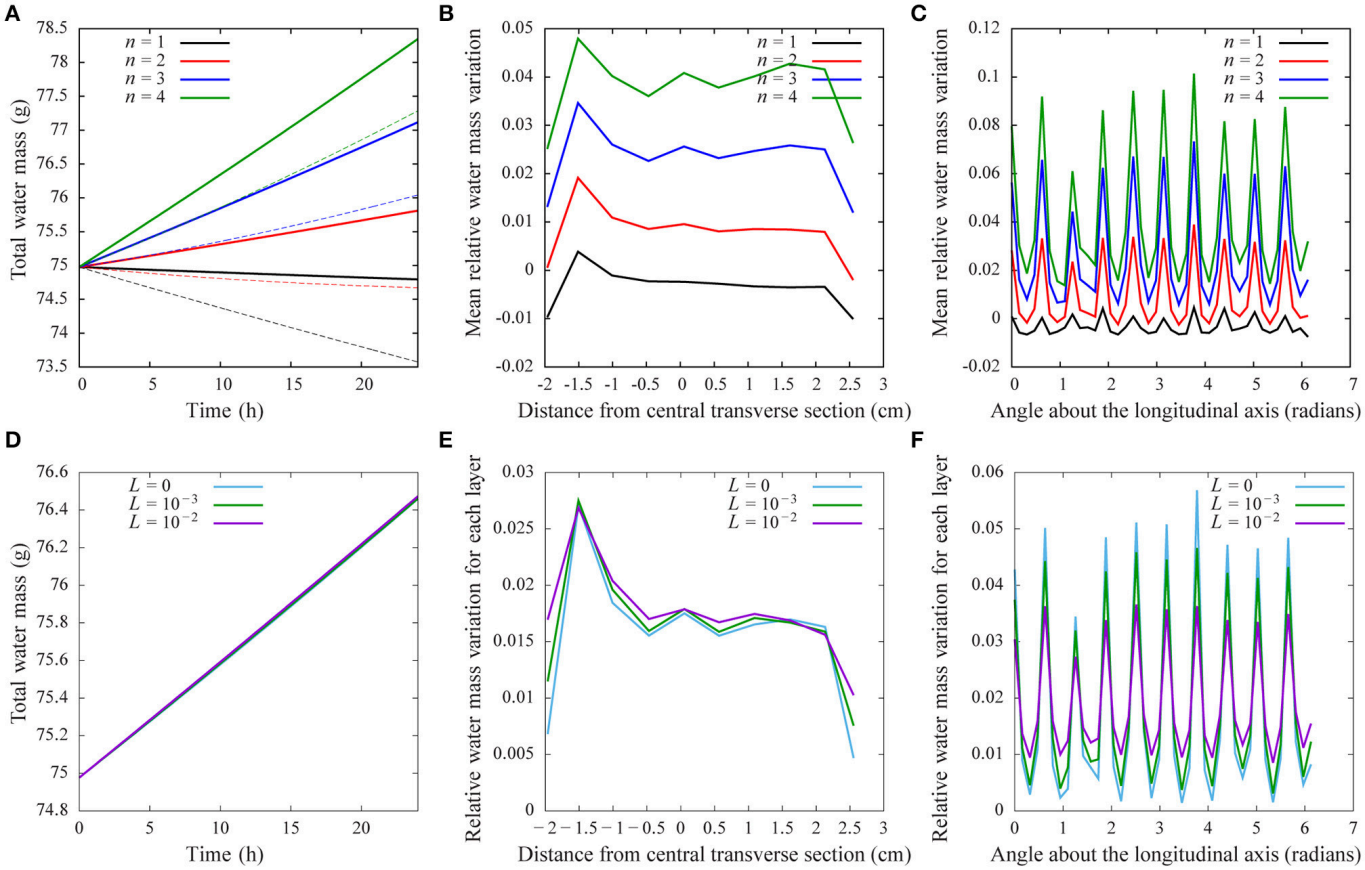
**Results of numerical simulations using the tomato fruit model: (A–C)** with different values of the pipe exponent and zero hydraulic conductivity between compartments (*L* = 0), and **(D–F)** with different values of hydraulic conductivity (*L*) and a constant pipe exponent. **(A,D)** Total water mass as a function of time; dashed lines in **(A)** show the results of a four-fold increase in transpiration. **(B,C,E,F)** Mean value of the relative water mass variation per day in the same layers as in Figure 5.

Using the model, we then studied how variations in these architectural parameters affect the distribution of water in the fruit over a 24-h growth period. Because the time period is relatively short, we used the unconstrained growth model, which is computationally efficient and makes it possible to explore large parts of the parameter space. For the initial conditions, we assumed a homogeneous distribution of sugar concentration and turgor in the pericarp. That is water and sugar contents in each tetrahedron were adjusted to match both the volume of the initial mesh and the prescribed initial concentration. The model parameters (Table 1) were set according to Liu et al. (2007), and the sugar concentration *S*_*i*_ remained stable at 2%. Figures 6, 7 show the results of numerical simulations for different values of the pipe exponent, with and without fluxes between tetrahedra, which emphasizes the effects of vasculature architecture. Fluxes of water reflect the hierarchy of vein distribution in the fruit: Fluxes are higher for well vascularized tissues and in regions with bigger vessels. This effect can be qualitatively seen in Figure 6, where more vessel surface area is available for water and dry matter flow near the pedicel. More quantitatively, Figure 7A shows that the total water varies linearly with time in the simulated 24 h time interval. For *n* = {2,3,4}, the growth rate is positive and increases regularly with *n*. However, in the case of *n* = 1, the fluxes from the vessels are not able to overcome transpiration, and the relative water mass variation per day is negative and decreases with time. We performed simulations with 4-fold higher transpiration rates (dashed lines in Figure 7A) and observed that, under higher rates, the water fluxes from the vessels are no longer able to overcome transpiration in the case of *n* = 2 as well. The results show that the model is able to capture quantitatively the balance between water supply in the fruit and water loss due to transpiration. The geometry of the vasculature itself is of key importance in such regulation, especially at the blossom end of the fruit. Its effect is largely due to the role of contact surfaces between vessel tissues and flesh in each compartment, as illustrated by the correlation between the contact area and the relative water mass variation, especially for *n* = {2,3}, for which the peak in relative water mass variation at −1.5 cm from the central layer (Figure 7B) coincides with the peak in contact area (Figure 5B). The same correlation is visible between relative water mass variation and longitudinal sections in Figures 5B, 7C (nine peaks are seen in both graphs).

**Table 1:** **Parameter values used for the tomato simulations**.

**Parameter**	**Symbol**	**Value**
Ratio of soluble sugar	*Z*	0.52 (dimensionless)
Gas constant	*R*	83 cm^3^ bar mol^−1^ K^−1^
Temperature	*T*	293.15 K
Water density	*D_*w*_*	1.0 g cm^−3^
Sugar density	*D_*s*_*	1.6 g cm^−3^
Water molar mass	*M_*w*_*	18 g mol^−1^
Sugar molar mass	*M_*s*_*	342.3 g mol^−1^
Non-sugar osmotic pressure in fruit	*π_*b*_*	5 bar
Non-sugar osmotic pressure in phloem	*$\pi_{b}^{p}$*	5 bar
Threshold value for pressure	*Y*	1 bar
Cell wall extensibility	ϕ	0.02 bar^−1^ h^−1^
Elastic modulus	ε	100.0 bar
Hydric potential of the stem	*Ψ_*stem*_*	−2.2 bar
Sugar concentration in the stem	*C_*stem*_*	0.2 g g^−1^
Relative humidity in fruit	*H_*f*_*	0.996 (dimensionless)
Relative humidity in air	*H_*air*_*	0.7 (dimensionless)
Permeation coefficient	ρ	22.0 cm h^−1^
Growth respiration coefficient	*q_*g*_*	0.22 (dimensionless)
Maintenance respiration coefficient	*q_*m*_*	0.00042 h^−1^
Temperature ratio of maintenance respiration	*q*_10_	1.4 (dimensionless)
Reflectivity coefficient of phloem membrane	*σ_*p*_*	1 (dimensionless)
Maximal rate of active uptake per unit of area	*ν_*max*_*	9.10^−5^ g h^−1^ m^−2^
Michaelis-Menten constant	*K_*m*_*	0.08 (dimensionless)
Phloem to fruit conductance	*L_*pf*_*	5.10^−4^ g h^−1^ m^−2^ bar^−1^
Xylem to fruit conductance	*L_*xf*_*	1.10^−5^ g h^−1^ m^−2^ bar^−1^
Hydraulic conductivity between cell walls	*L*	0, 10^−3^, 10^−2^ g h^−1^ m^−2^ bar^−1^

We also studied the effect of enhancing water fluxes between tetrahedral compartments by increasing the value of hydraulic conductivity, *L*, between them. We found that while the global growth remains the same (Figure 7D), the gap between maximum and minimum values decreases as *L* increases (Figures 7E,F). This can be seen, for example, by comparing the size of the peaks for *L* = 0 and *L* = 10^−2^ in Figure 7F. It shows that with increased hydraulic conductivity water distribution inside the fruit becomes more homogeneous.

**Figure 8:**
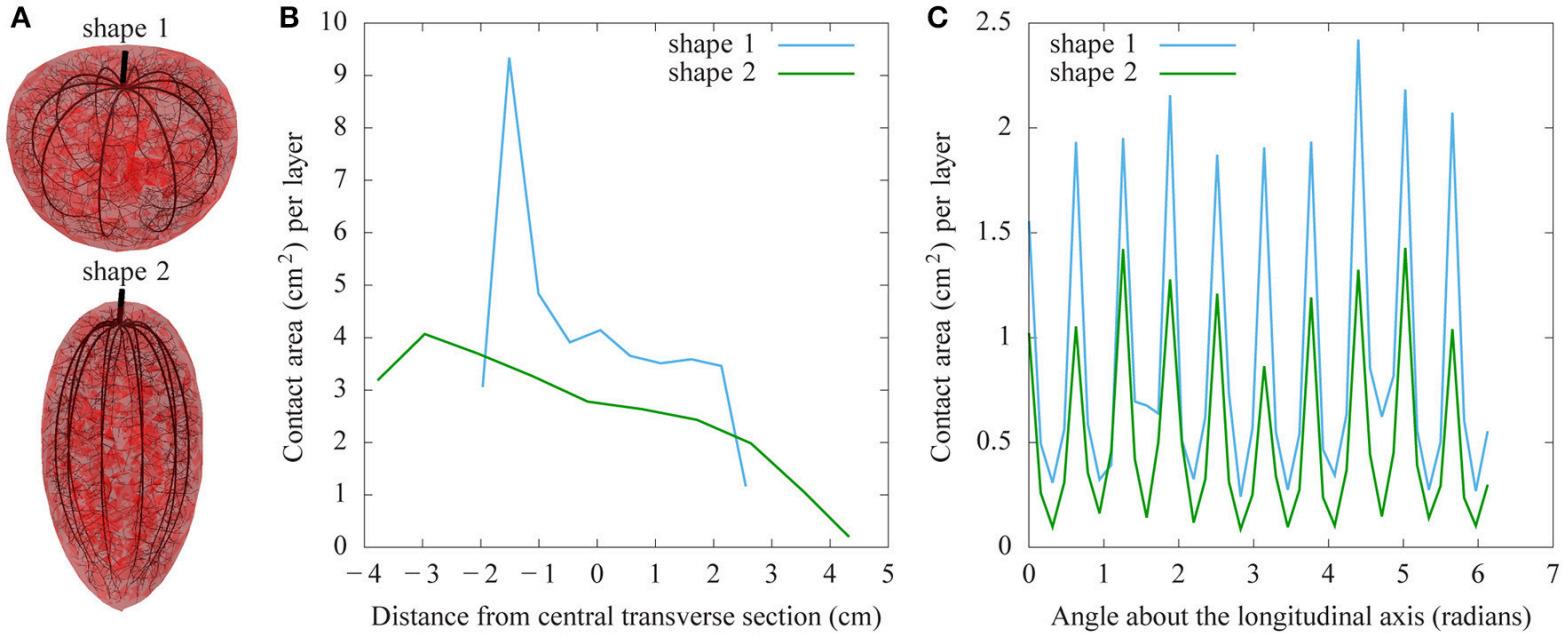
**Comparison of the effects of fruit shape on vascular patterns. (A)** Round tomato (shape 1) with the same pericarp volume as an elongated one (shape 2). For both shapes, the same density of attractor points was used to generate the vasculature. The plot shows the total lateral surface area of vessels in 10 transverse sections along the pedicel to blossom end **(B)** and in 40 longitudinal sections around the same axis **(C)**.

**Figure 9:**
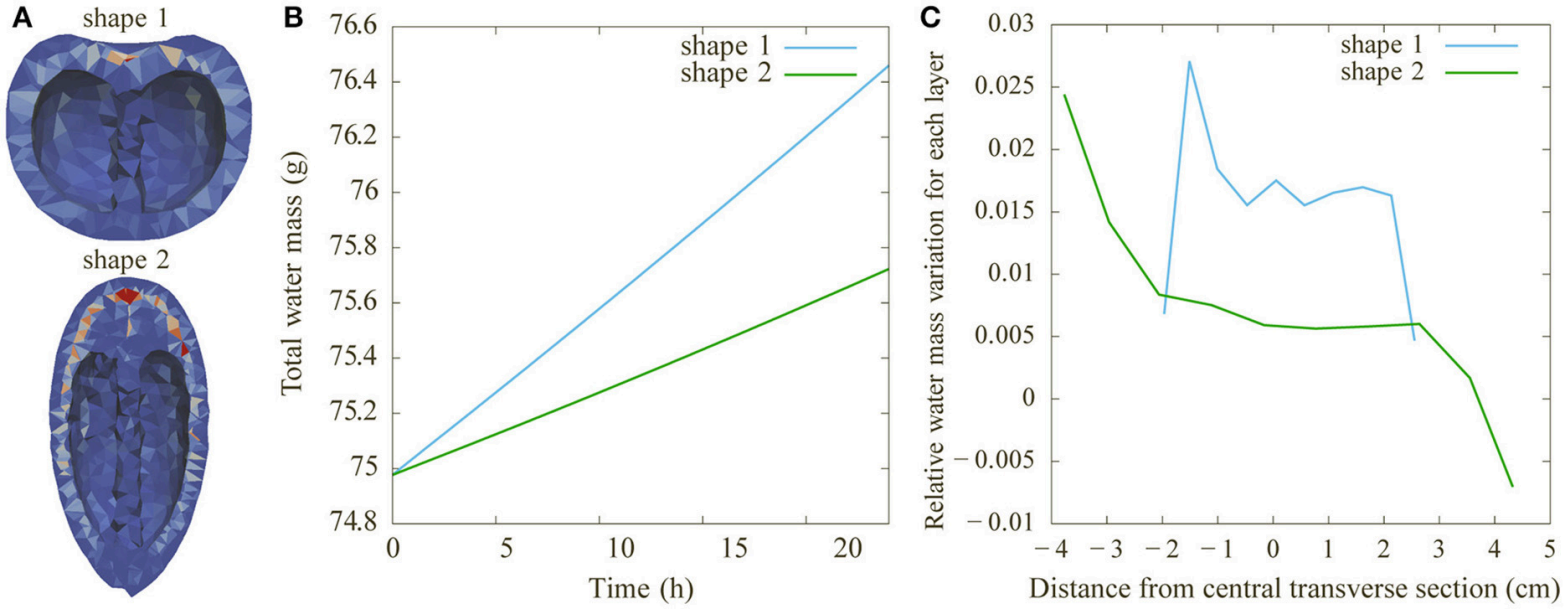
**Comparison of the effects of fruit shape on water distribution**. The comparison is between the two shapes from Figure 8 (round: shape 1; elongated: shape 2), with *n* = 2.5, and the same pericarp volume and density of attractor points. **(A)** Relative water mass variation as in Figure 6. **(B)** Total water mass as a function of time. **(C)** Mean value of the relative mass variation per layer along the pedicel to blossom end.

As the fruit shape itself is potentially a source of variability during fruit development, we compared the architecture of two tomato fruits with different shapes, but the same pericarp volume (Figure 8). The vasculature was computed in the two fruit types with exactly the same characteristics (same pedicel cross section at the origin, same number of main vessels, same density of attractor points for secondary vessels and same pipe coefficient: *n* = 2.5). The corresponding vascular patterns show a lower contact area at the blossom end of the elongated fruit. Figure 9 shows the resulting water distribution after a 24-h growth period. The relative water mass variation is much lower in the blossom end of the elongated fruit, even reaching negative values (due to transpiration). Therefore, under these assumptions, the round shape leads to a better hydration of the fruit. The water mass is greater and the relative hydration level is higher between vessels. Our results emphasize that fruit functioning and growth is sensitive to the shape of the fruit.

### Modeling the impact of microcrack formation on nectarine development and distribution of soluble sugars

In nectarine fruit, the spatial pattern of cuticular cracks on the fruit skin varies between the polar regions (stylar and peduncle ends) and equatorial regions (Gibert et al., 2007). This pattern of cracks changes the rate of water loss due to transpiration on different parts of the skin (Gibert et al., 2010). To analyse how such a pattern can cause a heterogeneous distribution of sugar inside a nectarine fruit, we used our functional-structural fruit model to perform simulations on the basis of our own experiments and on the work of Gibert et al. (2007, 2010). Since the heterogeneity in sugar distribution arises over time as microcracks develop, we used our fruit model to simulate nectarine growth with two additional aspects: (1) modeling constraints of growth and (2) modeling transpiration from microcracks in the fruit's skin. Although it is known that microclimate affects transpiration rates (Saudreau et al., 2007; Nordey et al., 2014), we assumed the temperature and humidity were uniform in the space surrounding the fruit. This simplification allowed us to concentrate on the effect of microcracks on sugar concentration and ignore other factors. The results were assessed against measurements of the percentage of microcracks on the skin and the sugar concentration in the mesocarp.

#### Measurements of microcracking and sugar concentration

**Figure 10:**
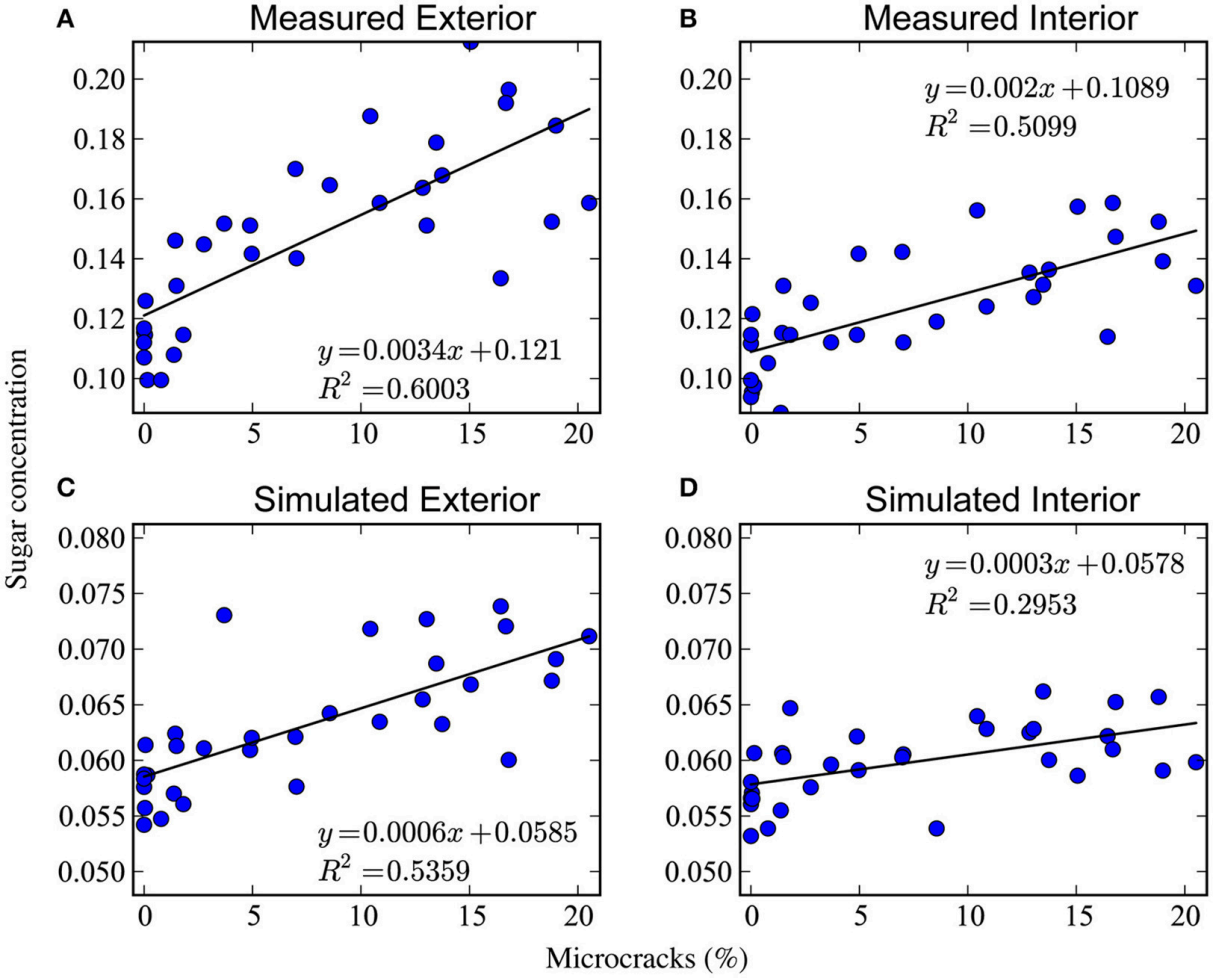
**Sugar content in a nectarine fruit as function of the percentage of microcracks at 140 dafb**. The two graphs on the top show the results of our measurements on the percentage of microcracks and sugar content (g soluble sugars/g FW) in the exterior region **(A)** and interior region **(B)** of a nectarine fruit. The two graphs on the bottom show the model output of sugar content (g soluble sugars/g FW) in the exterior region **(C)** and interior region **(D)**, which were obtained by modeling increased surface conductance due to microcracks.

The percentage of microcracks on the 32 measured regions of the fruit surface ranged from 0% to 20%. We measured a total fruit surface area of 150 cm^2^ of which 11.39 cm^2^ was cuticular microcracks (7.6%). For each of these 32 regions, the sugar content in the external section of the fruit's mesocarp ranged from 12 to 22 (Brix index), and in the internal section from 11 to 17 (Brix index). To facilitate comparison with model output, we converted the units of refractive index (Brix index) to sugar content (g soluble sugars/g FW). For the conversion we used the inverse of the relationship between refractive index and sugar content found by Grechi et al. (2008): $S^{*}=\left(RI-a_{r1}\right)/a_{r2}$, where S^*^ is the converted sugar content from our measured refractive index *RI* and the parameters *a*_*r*1_ = 79.41 (% g/g) and *a*_*r*2_ = 4.001 (%) are taken directly from the work of Grechi et al. (2008). The resulting sugar content ranged from 0.1 to 0.2 (g/g) in the external section and 0.1 to 0.15 (g/g) in the internal section of the mesocarp. Analysis of the data shows a linear relationship between the percentage of microcracks and sugar content in the 64 sections of the mesocarp (Figures 10A,B). Finally, the measured transpiration rate for the entire fruit was 4.8 × 10^−3^ g h^−1^ cm^−2^.

#### Simulations

**Figure 11:**
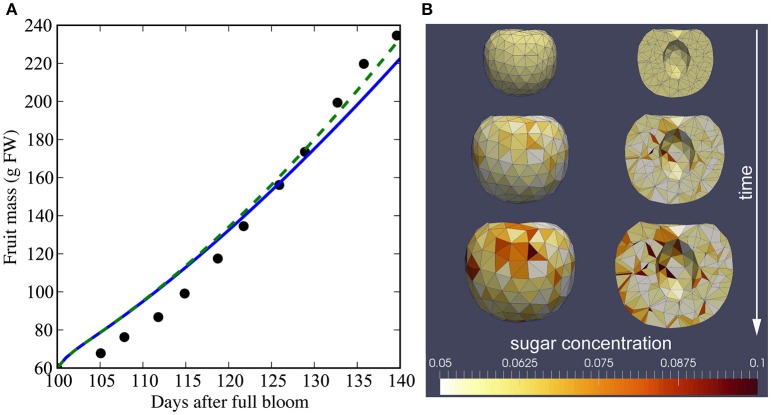
**Nectarine growth simulations. (A)** Simulated fresh weight of nectarine fruit growth with and without microcracks. The solid line shows fresh weight (g) with microcracks and the dashed line shows fresh weight (g) without microcracks. Black dots show fresh weight (g) as measured by Gibert et al. (2007). **(B)** Selected frames from the simulation at 100, 120, and 140 dafb. The tetrahedra are colored according to sugar concentration indicated by the color bar.

**Figure 12:**
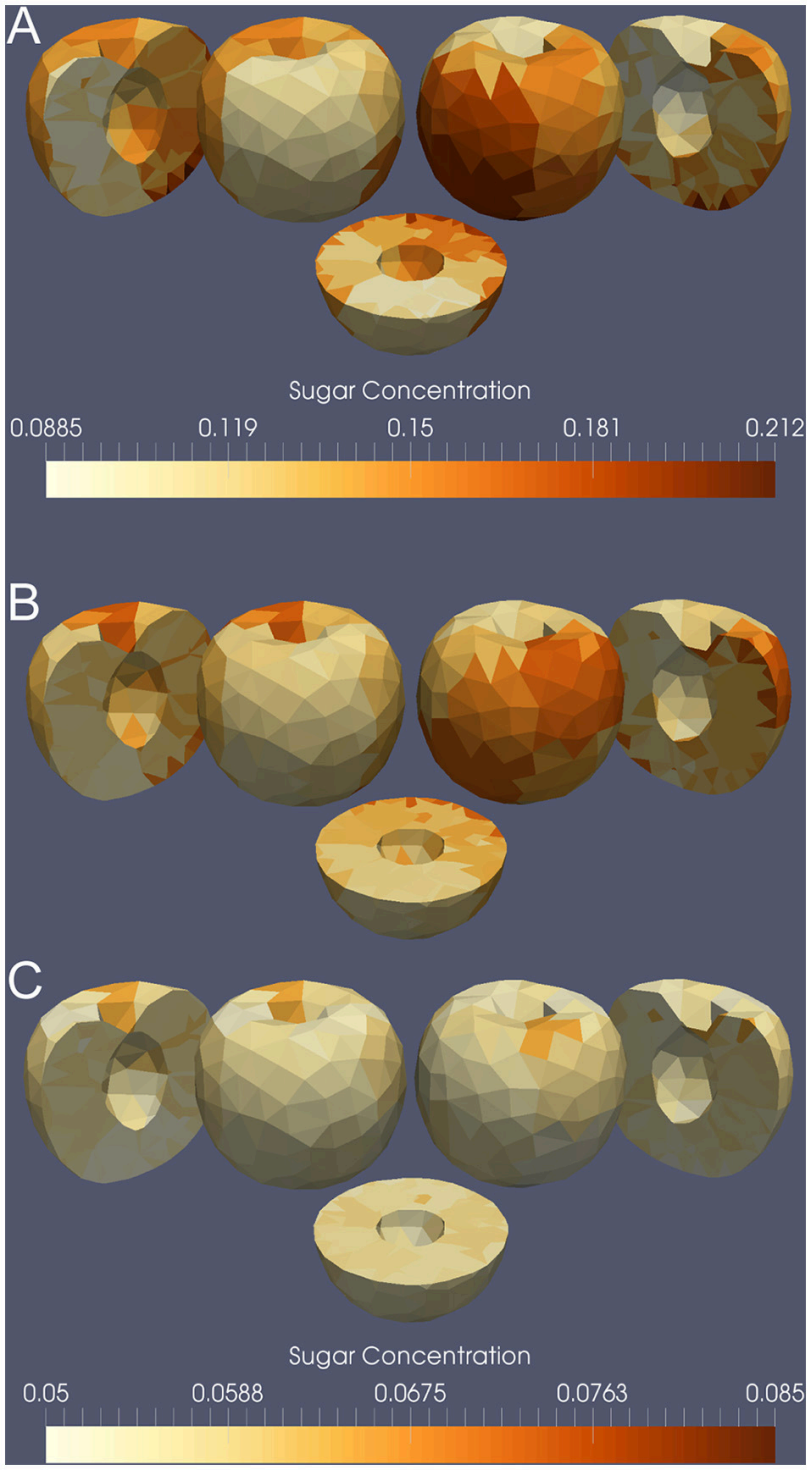
**A 3D visualization of sugar content in a nectarine fruit at 140 dafb**. The image on the top shows the measured data **(A)**, the image in the middle shows the simulated result with microcracking **(B)**, and the image on the bottom shows the simulated result without microcracking **(C)**. Each tetrahedron is colored according to the average sugar content (g soluble sugars/g FW) in its region of the fruit (which was divided into 32 interior and exterior regions). The visualization shows the whole fruit from the front and back, with corresponding longitudinal sections, and a transverse section. Dark brown indicates high sugar content while light yellow indicates low sugar content. The tetrahedra representing the stone are not shown.

The initial variable and parameter values used in our simulations are given in Table 2. The majority of these values were taken from a model of peach fruit growth (Fishman and Génard, 1998). We simulated growth from 100 to 140 days after full bloom (dafb), and set the initial fresh mass to 60 g (Gibert et al., 2007). The mesh had 1348 total tetrahedra (108 for the stone and 1240 for the mesocarp), with a mean initial volume of 4.3 × 10^−2^ cm^3^ and s.d. 1.3 × 10^−2^ cm^3^. The initial lateral area of vasculature in a compartment was set to $A_{i}^{v}\left(0\right)$ = 0.005 cm^2^ such that the final fresh mass of the fruit was 200–250 g. Figure 11A shows the model output for total fresh weight from a simulation of the last 40 days of growth after full bloom. The total fresh weight at time *t* was computed by summing the water and dry matter, *w*_*i*_(*t*) and *s*_*i*_(*t*), contents of each tetrahedral compartment *i* in the mesh. The total fruit surface area at 140 dafb was 185 cm^2^ with 15.6 cm^2^ cuticular microcrack area (8.4%). In simulations with and without microcracks the global behavior of the model reproduced qualitatively the observed pattern of growth for a low crop load study by Gibert et al. (2007). The simulated transpiration rate at 140 dafb was 2.6 × 10^−3^ g h^−1^ cm^−2^. Figure 11B shows selected time points from the simulation of nectarine growth. At the end of the simulation (140 dafb), the sugar concentrations of the 32 exterior and 32 interior regions of the fruit mesocarp were compared with our measured sugar content. The 3D mesh was divided into 64 regions, and we computed the average sugar concentration of the tetrahedra contained within each region. The model output showed a direct linear relationship between sugar content and the percentage of microcracks (Figures 10C,D). A 3D visualization of the measurements (Figure 12A) and the model output (Figure 12B) also shows a gradient of sugar from the interior to the exterior of the fruit. Furthermore, simulations of fruit growth with no microcracking resulted in a homogeneous distribution of sugar inside the fruit and a decrease in sugar content for the whole fruit (Figure 12C). This is in agreement with the observed outcome of covering fruit with clear plastic film (Li et al., 2001).

**Table 2:** **Parameter values used for the simulation of nectarine growth**.

**Parameter**	**Symbol**	**Value**
Ratio of soluble sugar	*Z*	0.61 (dimensionless)
Gas constant	*R*	83 cm^3^ bar mol^−1^ K^−1^
Temperature	*T*	293.15 K
Water density	*D_*w*_*	1.0 g cm^−3^
Sugar density	*D_*s*_*	1.6 g cm^−3^
Water molar mass	*M_*w*_*	18 g mol^−1^
Sugar molar mass	*M_*s*_*	342.3 g mol^−1^
Non-sugar osmotic pressure in fruit	*π_*b*_*	6.5 bar
Non-sugar osmotic pressure in phloem	*$\pi_{b}^{p}$*	12.53 bar
Threshold value for pressure	*Y*	5 bar
Cell wall extensibility	ϕ	0.01 bar^−1^ h^−1^
Elastic modulus	ε	1.0 bar
Hydric potential of the stem	*Ψ_*stem*_*	−6.0 bar
Sugar concentration in the stem	*C_*stem*_*	0.18 g g^−1^
Relative humidity in fruit	*H_*f*_*	0.996 (dimensionless)
Relative humidity in air	*H_*air*_*	0.7 (dimensionless)
Permeation coefficient	*ρ_*ext*_*	432.0 cm h^−1^
Permeation coefficient of microcracks	*ρ_*crk*_*	3838.0 cm h^−1^
Growth respiration coefficient	*q_*g*_*	0.21 (dimensionless)
Maintenance respiration coefficient	*q_*m*_*	0.000131 h^−1^
Temperature ratio of maintenance respiration	*q_10_*	2.03 (dimensionless)
Reflectivity coefficient of phloem membrane	*σ_*p*_*	0.9 (dimensionless)
Maximal rate of active uptake per unit of area	*ν_*max*_*	0.0031 g h^−1^ m^−2^
Michaelis-Menten constant	*K_*m*_*	0.08 (dimensionless)
Phloem to fruit conductance	*L_*pf*_*	0.05833 g h^−1^ m^−2^ bar^−1^
Xylem to fruit conductance	*L_*xf*_*	0.0128326 g h^−1^ m^−2^ bar^−1^
Hydraulic conductivity between cell walls	*L*	0, 0.005 g h^−1^ m^−2^ bar^−1^
Compartmental resistance to expansion	*λ_*v*_*	1
Noise control in Metropolis dynamics	*G*	10^−5^
Random step size	ϵ	0.01

## Discussion

We developed a functional-structural model of fruit development combining both architectural and physiological aspects. The model makes it possible to design a variety of fruit architectures by varying the envelope shape, the number of carpels, the thickness of the different tissue layers and the fruit vasculature in shape and density. These digital architectures can be generated either from measurements on real plants (shapes, densities of vessels, sizes of organs, etc.) or from synthetic data produced by geometric models. Water and sugar transport processes are modeled in a detailed manner based on the description of realistic vascular networks. These quantities participate in the regulation of fruit growth along with cell wall parameters such as elastic modulus, extensibility, and plastic expansion threshold. Fruit growth is also modulated by resource availability coming from other plant parts and by exogenous factors like temperature and humidity. As output, the model produces growth rates and physiological variables in space and time such as sugar content, turgor pressure, and water and dry matter fluxes. Different computation steps are necessary to run the model: (1) fruit mesh generation (2) vascular network generation, and (3) fruit functioning and growth specification. All these steps can be carried out on 3D growing fruits using a standard desktop computer in a limited amount of time (on the order of several hours). The model can be used to explore the impact of various architectural and biophysical traits on fruit quality. We demonstrated this by examining (1) the effects of patterns of vascular bundles on nutrient transport in tomato with different architectures, and (2) the role of skin microcracking on determining the distribution of sugar content in nectarine fruit.

Our model integrates the advantages of advanced process-based models, often used to study fruit growth in response to environmental factors (Fishman and Génard, 1998; Lechaudel et al., 2007; Liu et al., 2007), with a detailed description of fruit tissues and vascular architecture. In general, functional-structural models have been used to study whole-plant behavior and development, under different environmental conditions (Allen et al., 2005; Godin and Sinoquet, 2005; Vos et al., 2010) or to investigate local morphogenetic systems, describing the emergence of new plant organs at the cellular or tissue levels (Geitmann and Ortega, 2009; Merks et al., 2011; Prusinkiewicz and Runions, 2012; Boudon et al., 2015). Here we extend this approach to the description of the interior of an expanding organ. Morphogenesis is not modeled explicitly: As in the case of plant models, the initial architecture of the fruit tissues and vascular system are imposed to the model; cell division and differentiation phases are assumed to be completed, so that tissue identity remains stable over the entire simulation. Similarly, fruit microstructure is not explicitly modeled (Mebatsion et al., 2009; Fanta et al., 2014; Ho et al., 2016): Water and dry matter exchange between internal tetrahedra is represented as a simple diffusion process, with an effective homogeneous diffusion coefficient (Ho et al., 2010). The focus is set on the biophysical description of tissue expansion as driven by turgor pressure. Unlike most morphogenetic models, the main physicochemical processes underlying tissue expansion are described explicitly. This includes dry matter and water uptake, flows and distribution within the fruit architecture, turgor and osmotic pressure generation, carbon metabolism and the description of cell wall extensibility. Moreover, the effect of environmental conditions (temperature and humidity) onto water and dry matter distributions are accounted for, through their modulation of fruit respiration and transpiration processes. Yet, in the current version of the model, we did not consider an explicit modeling of the convection of the different elements in the vascular bundles (concentrations are assumed to propagate instantaneously inside the bundles). The dynamics of xylem and phloem flows will likely affect local water and dry matter exchange, contributing to the heterogeneity of composition within the fruit. Future models should take into account flux within vessels by means of fluid motion equation, as was done for blood in models of the cardiovascular system (Taylor et al., 1998; Mills et al., 2013).

Fruit shape, solid content, skin thickness, flesh fibrosity related to vasculature, or skin microcracks are important traits of quality that drive consumer purchasing and acceptance. These traits are usually manipulated individually through genetic selection or monitored through environmental and cultural practices. The present work highlighted their interconnections in the control of fruit growth and quality build-up. In particular the interaction between fruit shape and vessel density was shown to induce, independently of fruit size, an important and contrasted gradient of water supply along the vertical fruit section (from pedicel to blossom end) (Figure 9). The model predicted lower water supply to the tip end of elongated fruits (contact area close to zero, Figure 8), which is consistent with the sensitivity of elongated tomatoes to BER disorder. Indeed the weak vascular bundle development in the distal part of the fruit was suggested to promote Ca-related disorders (Ho and White, 2005) although calcium content *per se* may not be the only cause of BER development (de Freitas et al., 2011). Whereas, the genetic control of fruit shape is well-known (Causse et al., 2011), no gene controlling fruit vasculature development has been reported so far and such interaction between the two traits has been overlooked in genetic programs for quality. Yet, the conducting vessels have been extensively studied in plant tissues, as they are the main route for water and dry matter transport. In fleshy fruits, xylem and phloem anatomy has been assessed from fruit sections. In tomato, the number of vascular bundles increases during the period of rapid fruit expansion, while the density of bundles sharply falls in parallel to the decline of Ca concentration in the fruit tissue (Belda and Ho, 1993). The observations of Belda and Ho (1993) support the overall shape of the vessel architecture predicted in the present work. However, experimental data of the spatial arrangement of fruit vessels are currently missing and deserve more attention in the future. In a recent work (Herremans et al., 2015), the spatial development of transport structures was observed by X-ray microtomography in apple fruit, revealing a high degree of branching and an increase of the length of the vascular network from 5 to 20 m from 9 to 22 weeks after full bloom, corresponding to 5 cm of vascular tissue per cubic centimeter of apple tissue. Such insight into the fruit vasculature opens news perspectives for understanding and optimizing the interactions between fruit shape and vascular bundle development. Our work suggests that such traits may arise as important criteria in selection for fruit production under water limited environments, and that a 3D model may be useful to optimize fruit architecture in relation to other fruit traits and environmental factors.

Another important issue outlined by our 3D fruit model is the role of fruit skin in determining the heterogeneity in sugar content within the flesh. The fruit skin plays a prominent role in fruit growth, fruit cracking, fruit texture and protection against multiple biotic and abiotic stress factors (Bargel and Neinhuis, 2005; Saladie et al., 2007; Gibert et al., 2010). In the present work, fruit cracking was predicted with the model of Gibert et al. (2010) in order to match experimental measurements. Our simulated results show good agreement in the total fresh weight of the fruit, but a discrepancy in the sugar concentration. We speculate this is due to a lower transpiration rate in our model compared to the measured value (2.3 × 10^−3^ g h^−1^ cm^−2^ vs. 4.8 × 10^−3^ g h^−1^ cm^−2^). Because the cuticular and microcrack conductance values were taken from a different cultivar, it is possible that the transpiration rate would be higher for the cultivar in our study.

To test if increasing the transpiration rate in our model resolves the discrepancy, we ran additional simulations with increased surface conductance values (see Figure S1 in Supplementary Material). The results show that by increasing transpiration our model is able to reproduce the observed gradient in sugar concentration induced by microcracks. However, an amplifier must exist that accounts for the increase in transpiration rate. It is possible that microcrack density and local temperature are correlated. For example, if the microcracks were on the part of the fruit in full sun, transpiration would increase. Our model could simulate such an effect if the α term in Equation 13 was dependent on local temperature. In addition, Lescourret et al. (2001) observed considerable variation in surface conductance among fruits within the same peach cultivars, e.g., for “Suncrest” conductance ranged from 162 to 690 cm/h. Finally, we model the occurrence of cracks over time by considering the relative expansion rate of the cuticle as a function of fruit fresh mass and relative expansion rate of the fruit. The relationship can also vary within cultivars (Lescourret et al., 2001) and may depend on other factors, such as temperature.

The importance of skin properties and their variations at the fruit surface outline the need for a mechanistic model of fruit cracking (e.g., see models of fracture formation in tree bark Federl and Prusinkiewicz, 2004; Dale et al., 2014). Mechanical extensions of the model could allow us to investigate the impact of the stiffness of the fruit's skin on growth. This would also provide a method for simulating the formation of microcracks in response to stresses in the skin and to better account for the interactions and feedback loops between turgor-driven growth processes and cuticle mechanical constraints (Thompson, 2001; Bargel and Neinhuis, 2005). On the other hand, the microcrack-induced gradient of sugars within the fruit (Figure 12) may have several impacts. It may contribute to a negative taste perception, and it may also be involved in ripening heterogeneity or in the post-harvest evolution of fruit quality.

## Conclusion

Functional-structural fruit models integrating interactions and feedback loops between different tissues and processes are a promising tool to help investigate the interplay between genotype-specific architectural features and environmental factors in the control of fruit development, disease susceptibility, shelf life, and in the emergence of specific quality traits. Our work outlines the prominent role of the vascular network on fruit quality development, which may lead to innovative traits of selection for fruit production under stress prone environments. The fruit skin, which is already known to be a protective barrier, was shown to have a strong potential impact on sugar distribution. In the future, combining the functional-structural fruit model with a model simulating the microclimate surrounding the fruit would open the possibility for examining the effects of gradients in temperature (Saudreau et al., 2007; Nordey et al., 2014) and humidity (Li et al., 2001) on fruit quality development.

## Author contributions

FB, CG, MG, VB, and NB participated in the conception and design of the work, the supervision of MC and IC, the interpretation of data, and the writing and critical revision of the manuscript. NB performed the experiments. MC and IC performed the modeling work and the simulations, and participated in the writing and critical revision of the manuscript. All authors read and approved the final manuscript.

### Conflict of interest statement

The authors declare that the research was conducted in the absence of any commercial or financial relationships that could be construed as a potential conflict of interest.
