# Supplementary material for: Integrating Physiology and Architecture in Models of Fruit Expansion
Source: Front Plant Sci. 2016 Nov 21;7:1739. doi: 10.3389/fpls.2016.01739 (PMC5116533; doi:10.3389/fpls.2016.01739)
Supplement: Supplementary file 1 [file Image1.PDF]

Supplementary Material

INTEGRATING PHYSIOLOGY AND ARCHITECTURE IN MODELS OF FRUIT EXPANSION

Mikolaj Cieslak, Ibrahim Cheddadi, Frédéric Boudon, Valentina Baldazzi, Michel Génard, Christophe Godin, and Nadia Bertin*

*** Correspondence:** Corresponding Author: nadia.bertin@avignon.inra.fr

1 Supplementary Figures

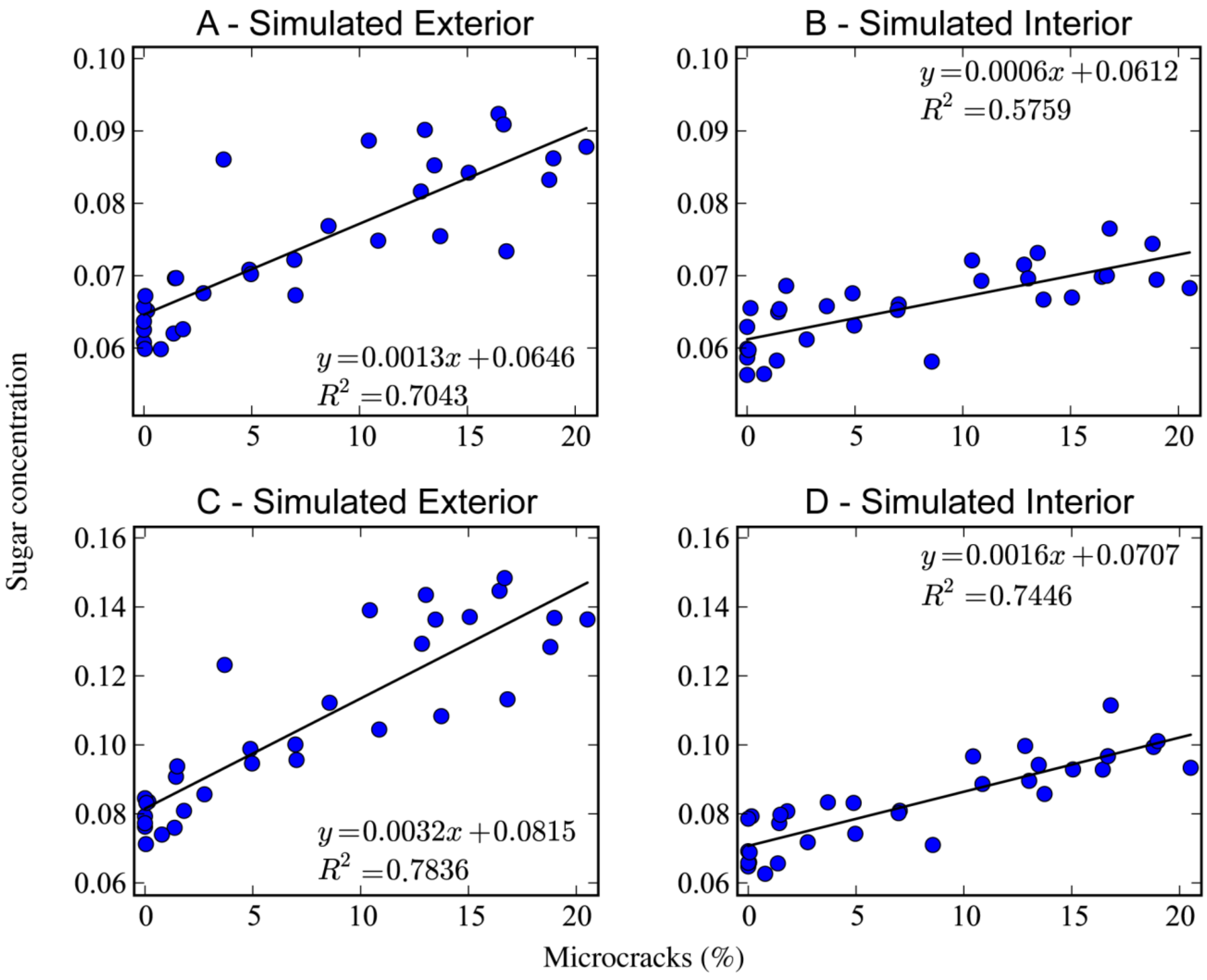

Supplementary Figure 1. Simulated sugar concentrations in a nectarine fruit at 140 dafb, as in Fig. 10, but with increased transpiration rates. (A,B) The transpiration rate was increased by two and (C,D) by five compared to the simulations presented in the Results section of the main text. The slope of the line relating microcracks to sugar concentration is in better agreement with the measurements as the transpiration increases.
